# Augmented EPR effect post IRFA to enhance the therapeutic efficacy of arsenic loaded ZIF-8 nanoparticles on residual HCC progression

**DOI:** 10.1186/s12951-021-01161-3

**Published:** 2022-01-15

**Authors:** Xuehua Chen, Yongquan Huang, Hui Chen, Ziman Chen, Jiaxin Chen, Hao Wang, Dan Li, Zhongzhen Su

**Affiliations:** 1 Department of Ultrasound, Fifth Affiliated Hospital of Sun Yat-sen University, Zhuhai, 519000 Guangdong China; 2 Guangdong Provincial Key Laboratory of Biomedical Imaging and Guangdong Provincial Engineering Research Center of Molecular Imaging, Fifth Affiliated Hospital of Sun Yat-sen University, Zhuhai, 519000 Guangdong China; 3 Fine Chemical Industry Research Institute, School of Chemistry, Sun Yat-sen University, Guangzhou, 510275 Guangdong China

**Keywords:** Hepatocellular carcinoma, Incomplete radiofrequency ablation, Arsenic trioxide, Antitumor, ZIF-8

## Abstract

**Background:**

Insufficient radiofrequency ablation (IRFA) can promote the local recurrence and distal metastasis of residual hepatocellular carcinoma (HCC), which makes clinical treatment extremely challenging. In this study, the malignant transition of residual tumors after IRFA was explored. Then, arsenic-loaded zeolitic imidazolate framework-8 nanoparticles (As@ZIF-8 NPs) were constructed, and their therapeutic effect on residual tumors was studied.

**Results:**

Our data showed that IRFA can dramatically promote the proliferation, induce the metastasis, activate the epithelial–mesenchymal transition (EMT) and accelerate the angiogenesis of residual tumors. Interestingly, we found, for the first time, that extensive angiogenesis after IRFA can augment the enhanced permeability and retention (EPR) effect and enhance the enrichment of ZIF-8 nanocarriers in residual tumors. Encouraged by this unique finding, we successfully prepared As@ZIF-8 NPs with good biocompatibility and confirmed that they were more effective than free arsenic trioxide (ATO) in sublethal heat-induced cell proliferation suppression, apoptosis induction, cell migration and invasion inhibition, and EMT reversal in vitro. Furthermore, compared with free ATO, As@ZIF-8 NPs exhibited remarkably increased therapeutic effects by repressing residual tumor growth and metastasis in vivo.

**Conclusions:**

This work provides a new paradigm for the treatment of residual HCC after IRFA.

**Graphical Abstract:**

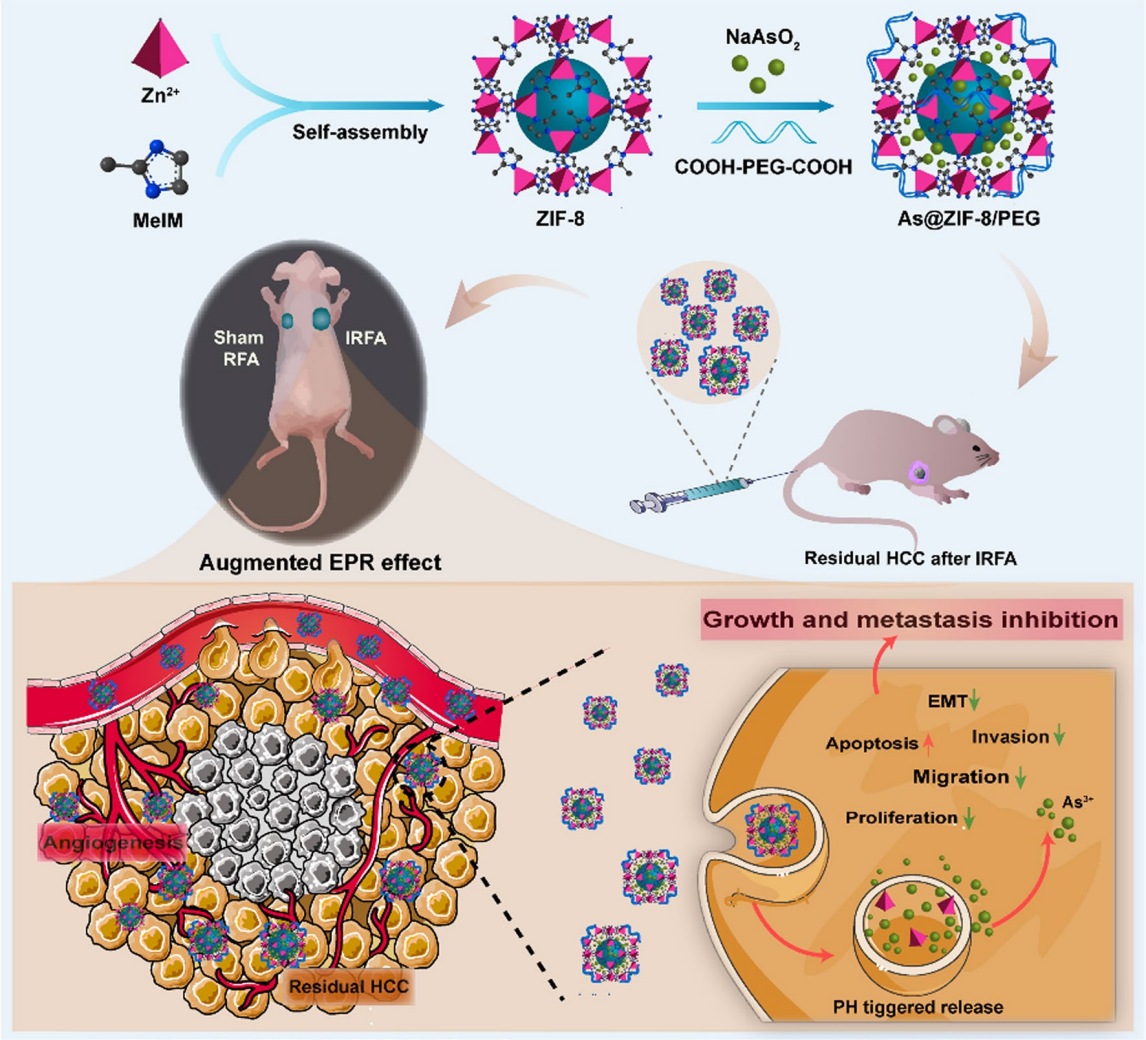

**Supplementary Information:**

The online version contains supplementary material available at 10.1186/s12951-021-01161-3.

## Introduction

Hepatocellular carcinoma (HCC) is the sixth most common cancer, and its mortality rate is third among cancer-related deaths worldwide [1]. Radiofrequency ablation (RFA) is a curative therapy for early HCC that causes thermal ablation at the tumor location and induces local coagulative necrosis of tumor cells [2–4]. As a minimally invasive treatment, RFA exhibits the advantages of simplicity, safety, short hospitalization and few complications [5]. However, the local recurrence rate of HCC after RFA ranges from 2 to 36% [6]. Moreover, recurrent tumors tend to have a high malignant grade and show low sensitivity to traditional chemotherapy, which ultimately leads to a worse patient prognosis [5]. These findings may be primarily attributed to the residual tumor that remains after insufficient radiofrequency ablation (IRFA). Multiple mechanisms have been reported to endow post-IRFA residual tumors with aggressive phenotypes, including enhanced proliferative ability [7], heightened angiogenetic capacity [8, 9], activated epithelial-to-mesenchymal transition (EMT) [3, 10] and increased migratory and invasive potential of tumor cells [11, 12]. Moreover, emerging evidence has shown that these multiple complicated processes can influence each other and together drive local recurrence and distant metastasis of the residual tumor and induce cancer cell resistance to traditional chemotherapy drugs, which makes clinical treatment extremely challenging [8, 13–15].

Arsenic trioxide is the most bioactive single-drug treatment used in the intervention of acute promyelocytic leukemia (APL) [16]. It has also been recommended as a frontline therapy in advanced HCC by the Chinese Society of Clinical Oncology. Recent studies have demonstrated that ATO can suppress HCC cells through various mechanisms in vitro, such as suppressing proliferation [17, 18], retarding invasion and migration [19, 20], and reversing multidrug resistance [21, 22]. These versatile effects imply that ATO may be suitable for the eradication of residual tumor cells. However, the poor bioavailability and narrow drug safety window of ATO severely restrict its clinical applications [23]. Specifically, as a water-soluble drug, ATO shows low delivery efficiency and rapid renal elimination, which inhibit its accumulation in the tumor site [24–26]. Therefore, to achieve the desired therapeutic effect, a large dose of ATO is required, but this high dose can lead to serious hepatotoxicity and nephrotoxicity [23, 26]. Although some improved measures, such as intra-arterial infusion and arsenic efflux micropumps, have been used to minimize side effects, the overall survival of patients with HCC after ATO treatment has not been significantly prolonged [25]. Therefore, the development of a novel method is urgently needed to increase drug accumulation in residual HCC sites and increase its therapeutic efficacy while maintaining high biocompatibility.

In recent years, the rapid development of nanomedicine has led to great opportunities to improve the bioavailability of chemotherapeutic drugs and reduce their systemic toxicity. Based on the enhanced permeability and retention (EPR) effect, arsenical nanoparticles (NPs) can be selectively enriched in tumor sites to minimize side effects and maximize therapeutic effects [20]. A few ATO-encapsulated organic- and inorganic-based drug delivery systems have been designed for the treatment of tumors, and these systems include nanoliposomes [27, 28], mesoporous hollow zirconia spheres and silica NPs [20, 29]. More encouragingly, arsenene, which is a two-dimensional nanomaterial composed of monoelemental arsenic, shows a suitable moderate band structure, high carrier mobility, and good optical properties [30]. Some recent studies have confirmed that arsenene can be applied not only as an intrinsic transformative valence-activated chemotherapy drug to produce large amounts of reactive oxygen species, but also as a photothermal therapy agent and an in vivo imaging agent, which greatly broadens the biomedical application of arsenical drugs [31, 32]. However, the therapeutic compounds inside these liposomes easily leak out before reaching the target site [33]. The biodegradation process of mesoporous zirconia hollow spheres, silica NPs and newly reported arsenene in vivo remains unclear. Metal–organic frameworks (MOFs) is a family of crystalline porous composites constructed by organic ligand self-assembly with metal nodes/metal clusters, which has attracted extensive attention in recent years [34–39]. Due to their component and structural variability, large surface areas with high porosity, and safe biodegradability, MOFs have been recently studied as promising nanocarriers for biomedical applications [34]. Zeolitic imidazolate framework-8 (ZIF-8), an MOF, is generate from zinc ions and 2-methylimidazole [40]. In addition to the characteristics of MOFs, ZIF-8 NPs possess ideal thermal and hydrothermal stabilities [41]. Furthermore, ZIF-8 NPs can decompose into zinc ions and imidazolate ions in the pH range of 5.0–6.0, which indicates that these materials are ideal nanocarriers for pH-responsive drug delivery and biodegradation in the acidic tumor microenvironment [42]. Importantly, zinc ions and imidazolate ions are nontoxic constituent elements of physiological systems: zinc is the second most essential trace metal element in the human body, and the imidazole group is a component of histidine [43]. However, nanoparticles with large surface areas always tend to aggregate and adsorb plasma proteins when injected into animals, which makes them rapidly cleared by macrophages [44]. One possible solution is to modify polyethylene-glycol (PEG) on the surface of ZIF-8, which helps provide greater colloidal stability, minimize protein adsorption, reduce uptake by the reticuloendothelial system and prolong the blood circulation time of nanoparticles in the bloodstream [44, 45]. Recently, ZIF-8 nanocarriers were used to deliver doxorubicin for chemotherapy [41, 42]; these carries biomineralize for the efficient delivery and release of proteins [46] and can accommodate loaded DNA enzymes for gene therapy [47], which confirms their great application potential. However, few studies have investigated the therapeutic effect and potential mechanism of arsenic-based ZIF-8 NPs in the treatment of residual HCC after IRFA.

In this study, we developed ZIF-8-based NPs to deliver ATO and originally hypothesized that substantial levels of angiogenesis post-IRFA can augment the EPR effect and thus enhance the accumulation of NPs in residual tumors. Then, we evaluated the bioavailability and explored the efficacy of these NPs in inhibiting cell proliferation, inducing apoptosis, decreasing migration and invasion, and suppressing EMT of residual tumor cells in vitro using a sublethal heat model. Additionally, tumor xenografts and lung metastasis models were employed to assess the efficacy on residual tumor growth, EMT and cancer cell metastasis in vivo. Overall, this novel treatment strategy improves therapeutic effects and is highly promising for use in residual HCC therapy.

## Results

### IRFA promoted the growth, metastasis, EMT and angiogenesis of residual HCC

**Fig. 1:**
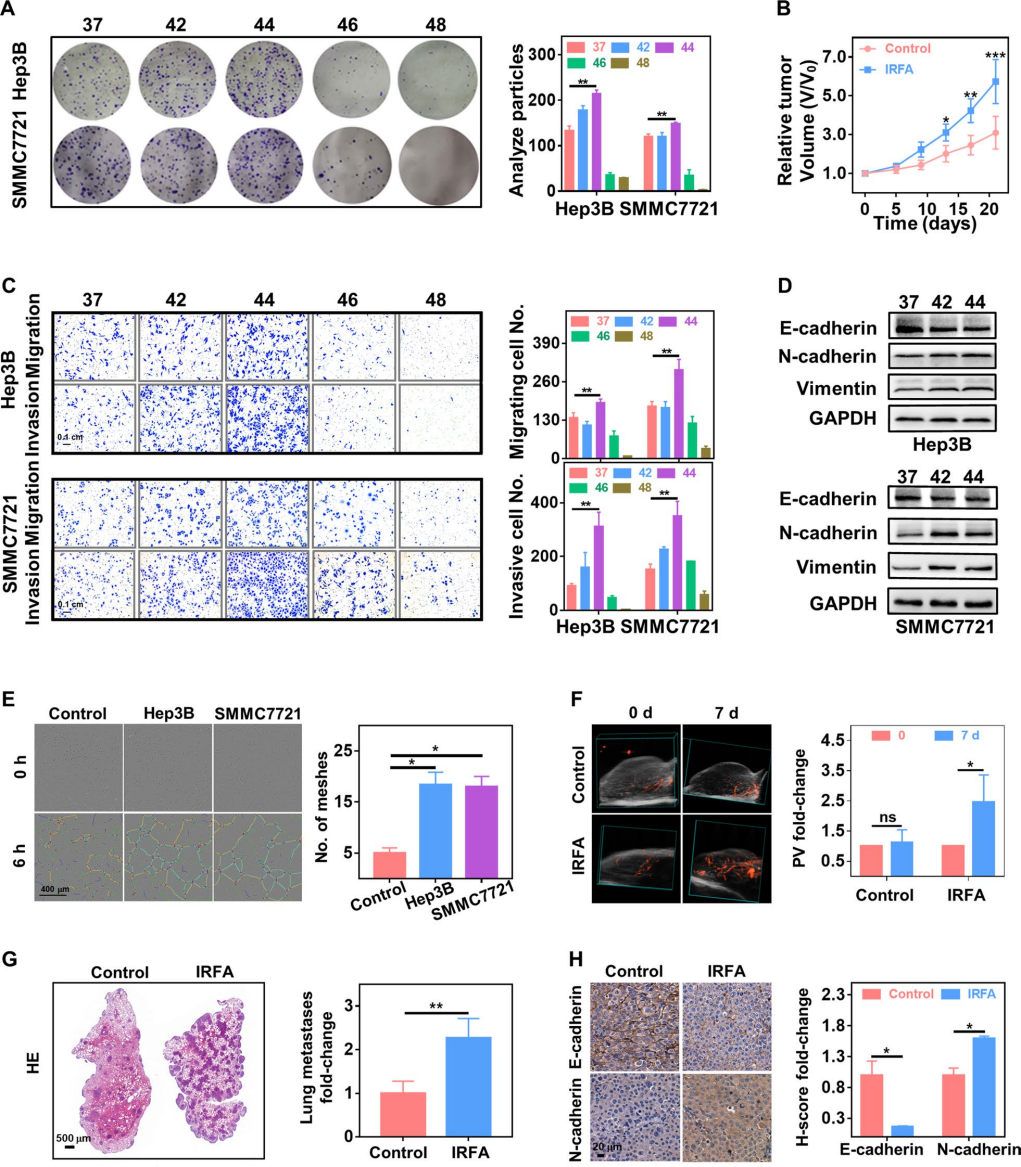
IRFA promoted the growth, metastasis, EMT and angiogenesis of residual HCC tumors. **A** Colony forming ability of Hep3B and SMMC7721 cells after heating at different temperatures for 15 min and a quantitative analysis chart (n = 3). **B** Growth curves of subcutaneous tumors with or without IRFA (n = 5). **C** Migration and invasion of Hep3B and SMMC7721 cells after heating at different temperatures for 15 min and a quantitative analysis chart (n = 3). Scale bar, 0.1 cm. **D** Protein expression of EMT markers vimentin, N-cadherin and E-cadherin in Hep3B and SMMC7721 cells after heating at different temperatures for 15 min. **E** Tube formation of HUVECs after coculture with or without the supernatant of sublethally heated Hep3B and SMMC7721 cells and quantification of tubule meshes (n = 3). Scale bar, 400 µm. **F** Three-dimensional power Doppler imaging of subcutaneous tumors with or without IRFA and the quantified percentage of blood vessels (PV) (n = 4). **G** HE staining of lung sections showing lung metastasis and the quantification analysis (n = 5). Scale bar, 500 µm. **H** Immunohistochemical staining of N-cadherin and E-cadherin in tumor tissues 21 days after IRFA or sham IRFA and the quantified H-score (n = 5). H-score, histochemistry score. Scale bar, 20 µm. (ns, no statistical difference; *P < 0.05; **P < 0.01; ***P < 0.001)

To mimic IRFA in vitro, we exposed Hep3B and SMMC7721 cells to different temperatures, namely, 42, 44, 46, and 48 °C, for 15 min. Cell viability was significantly decreased within 24 h of exposure to temperatures of 46 and 48 °C (Additional file 1: Fig. S1). Morphologically, the cells exposed to 44 °C exhibited spindle shapes 3 days after heating, and heat treatment at 46 and 48 °C induced vacuolar changes (Additional file 1: Fig. S2). Colony formation assays revealed that cell proliferation was more clearly enhanced by exposure to 44 °C than 42 °C, but increasing the temperature to 46 and 48 °C led to an inhibitory effect (Fig. 1A). Transwell assays indicated that upon heat treatment at 44 °C, the migration rate of Hep3B and SMCC7721 cells was increased by 35.91 ± 5.94% and 70.82 ± 11.70%, respectively, and the invasion rate was increased by 247.30 ± 35.34% and 131.70 ± 21.56%, respectively. Nevertheless, cell exposure to temperatures of 46 and 48 °C resulted in decreases in the migratory and invasive abilities of the cells (Fig. 1C). Based on these results, 44 °C was used as the optimal sublethal heating temperature for Hep3B and SMMC7721 cells in subsequent experiments. Because the EMT is implicated in tumor migration and invasion, western blot was performed to evaluate changes in this dynamic process after heat treatment. The results showed that heat treatment increased the levels of N-cadherin and vimentin and decreased the level of E-cadherin, which suggested that heat stress can promote the EMT process (Fig. 1D). Angiogenesis plays a key role in the rapid growth and metastasis of tumors. A tube formation assay was performed to assess whether IRFA promotes the angiogenesis potential of residual tumors. The results verified the finding that heat treatment enhanced the tube formation ability of human umbilical vein endothelial cells (HUVECs) in the HCC cell coculture system (Fig. 1E, Additional file 1: Fig. S3). Then, a subcutaneous IRFA tumor model (Additional file 1: Fig. S4) was successfully established under ultrasound guidance to evaluate the influence of IRFA on the growth and vascular distribution in residual cancer. The results showed that the growth of remnant cancer was significantly accelerated (Fig. 1B). Furthermore, power Doppler revealed that the percentage of blood vessels (PV) in residual tumors after IRFA was 2.47 ± 0.45-fold greater than that before ablation, but the PV in the control group did not change significantly (Fig. 1F). In addition, immunohistochemical staining of the vascular endothelial cell marker CD34 revealed higher numbers of blood vessels at the ablation boundary in the IRFA group, whereas sporadic blood vessels were observed under the tumor capsule in the control group (Additional file 1: Fig. S5). A lung metastasis model was also employed to determine the effect of IRFA on tumor metastasis in vivo, and the results showed more lung metastases in the IRFA group (Fig. 1G). Similarly, EMT marker staining of subcutaneous residual cancer tissue revealed that IRFA activated the expression of N-cadherin and suppressed the expression of E-cadherin (Fig. 1H). Collectively, these results suggested that IRFA promoted the proliferation, metastasis, EMT and angiogenesis of residual tumors after IRFA both in vitro and in vivo.

### Preparation and characterization of the arsenic loaded ZIF-8 NPs

**Fig. 2:**
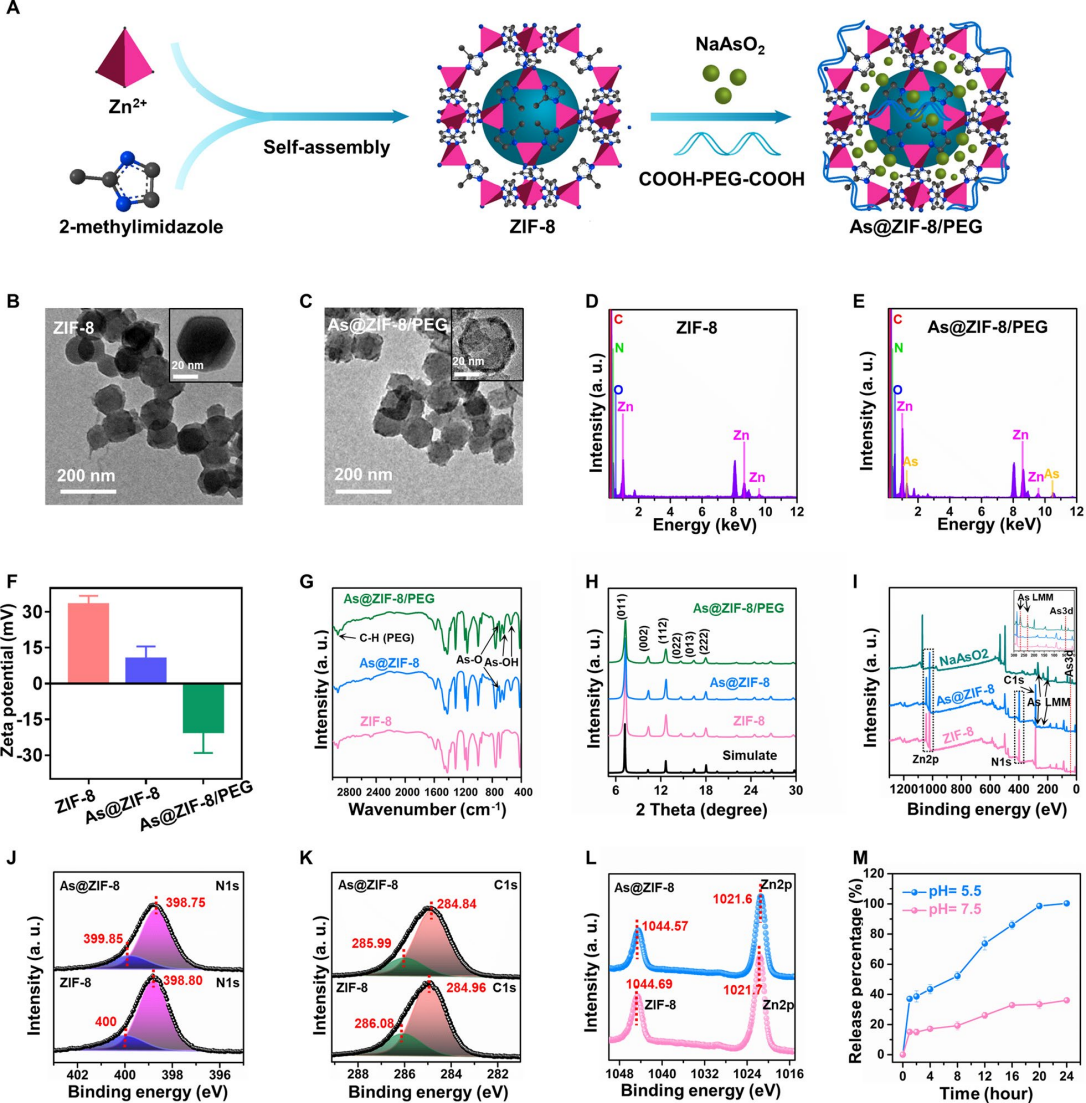
Preparation and characterization of ZIF-8, As@ZIF-8 and As@ZIF-8/PEG. **A** Schematic diagram of As@ZIF-8/PEG preparation. **B**, **C** TEM images of ZIF-8 (**B**) and As@ZIF-8/PEG (**C**). **D**, **E** TEM-EDS characterization of ZIF-8 (**D**) and As@ZIF-8/PEG (**E**). **F** Zeta potential of ZIF-8, As@ZIF-8, and As@ZIF-8/PEG (n = 3). **G** FTIR spectra of blank ZIF-8 (pink), As@ZIF-8 (blue), and As@ZIF-8/PEG (green). **H** PXRD spectra of simulated ZIF-8 (blank), ZIF-8 (pink), As@ZIF-8 (blue), and As@ZIF-8/PEG (green). **I**–**L** XPS of the as-prepared samples: XPS survey spectra of ZIF-8 (pink), As@ZIF-8 (blue) and NaAsO_2_ (green) (**I**); N (**J**), C (**K**) and Zn (**L**) XPS spectra of ZIF-8 and As@ZIF-8. **M** Cumulative release of As from As@ZIF-8/PEG at pH 5.5 and 7.4 (n = 2)

The synthetic procedure of As@ZIF-8/PEG is illustrated in Fig. 2A. The ZIF-8 nanocarrier was first prepared by mixing Zn(NO_3_)_2_·6H_2_O and 2-methylimidazole in methanol. Then, NaAsO_2_ and COOH–PEG–COOH were loaded onto ZIF-8 in ultrapure water to successfully synthesize As@ZIF-8/PEG. TEM analysis indicated the successful fabrication of polyhedral ZIF-8 NPs (Fig. 2B) with an average diameter of 75.13 ± 1.22 nm (Additional file 1: Fig. S6A). After the loading of arsenic and PEG, no significant changes in the morphology or particle size were observed (Fig. 2C, Additional file 1: Fig. S6B) and EDS confirmed the encapsulation of NaAsO_2_ into ZIF-8 nanocarriers (Fig. 2D, E). Moreover, the zeta potential of ZIF-8 changed from 33.02 ± 1.54 mV to − 20.12 ± 3.99 mV (Fig. 2F), which was mainly attributed to the encapsulation of negatively charged ^–^COO–PEG–COO^−^ and AsO_2_^−^. It can be inferred that the loading mechanism of NaAsO_2_ in As@ZIF-8 NPs may be electrostatic adsorption. Similarly, the FTIR spectra of AS@ZIF-8/PEG showed four additional peaks at 713, 641, 540 and ~ 2900 cm^−1^, which may be attributed to the vibrations of As–O, As–OH and C–H in PEG (Fig. 2G) [48, 49]. Moreover, the powder XRD results verified that the characteristic peaks of the prepared nanoparticle materials (ZIF-8, As@ZIF-8 and AS@ZIF-8/PEG) corresponded highly with simulated ZIF-8, illustrating that the crystal structure of ZIF-8 was not notably changed after modification (Fig. 2H) [47]. XPS was used to identify the atomic states of the samples. The dissimilarities between the XPS survey spectra of ZIF-8 and As@ZIF-8 centered mainly on As 3d peaks (44.3 eV) and As LMM peaks (268.23 eV) (Fig. 2I), which affirmed the successful accumulation of arsenic [50]. Correspondingly, the N 1 s peaks (Fig. 2J), C 1 s peaks (Fig. 2K), Zn 2p_3/2_ peaks (Fig. 2L) and Zn 2p_1/2_ peaks (Fig. 2L) slightly shifted toward lower binding energies, which indicates that the enhanced electron cloud density is relative to pure ZIF-8. The loaded arsenic was quantitatively analyzed by ICP-OES. The ratio of zinc to arsenic was as high as 3.3:1, which is equivalent to 86 mg of arsenic in 1 g of As@ZIF-8/PEG NPs.

To explore the drug release characteristics of AS@ZIF-8/PEG, we quantified the arsenic release profile at pH 5.5 and 7.5, which partially represent the acidic tumor microenvironment and physiological blood conditions, respectively. The results showed that the release of arsenic at pH 5.5 was more rapid and completed after 24 h, whereas at this time point, only 36.06 ± 1.25% of arsenic was released under neutral conditions (Fig. 2M). This superior pH-dependent release property of As@ZIF-8/PEG makes it possible to become a drug release system with the merits of accelerated drug release in the acidic tumor microenvironment and reduced toxicity to normal tissues and organs during circulation.

### As@ZIF-8 NPs inhibited the proliferation and promoted the apoptosis of sublethally heated HCC cells in vitro

**Fig. 3:**
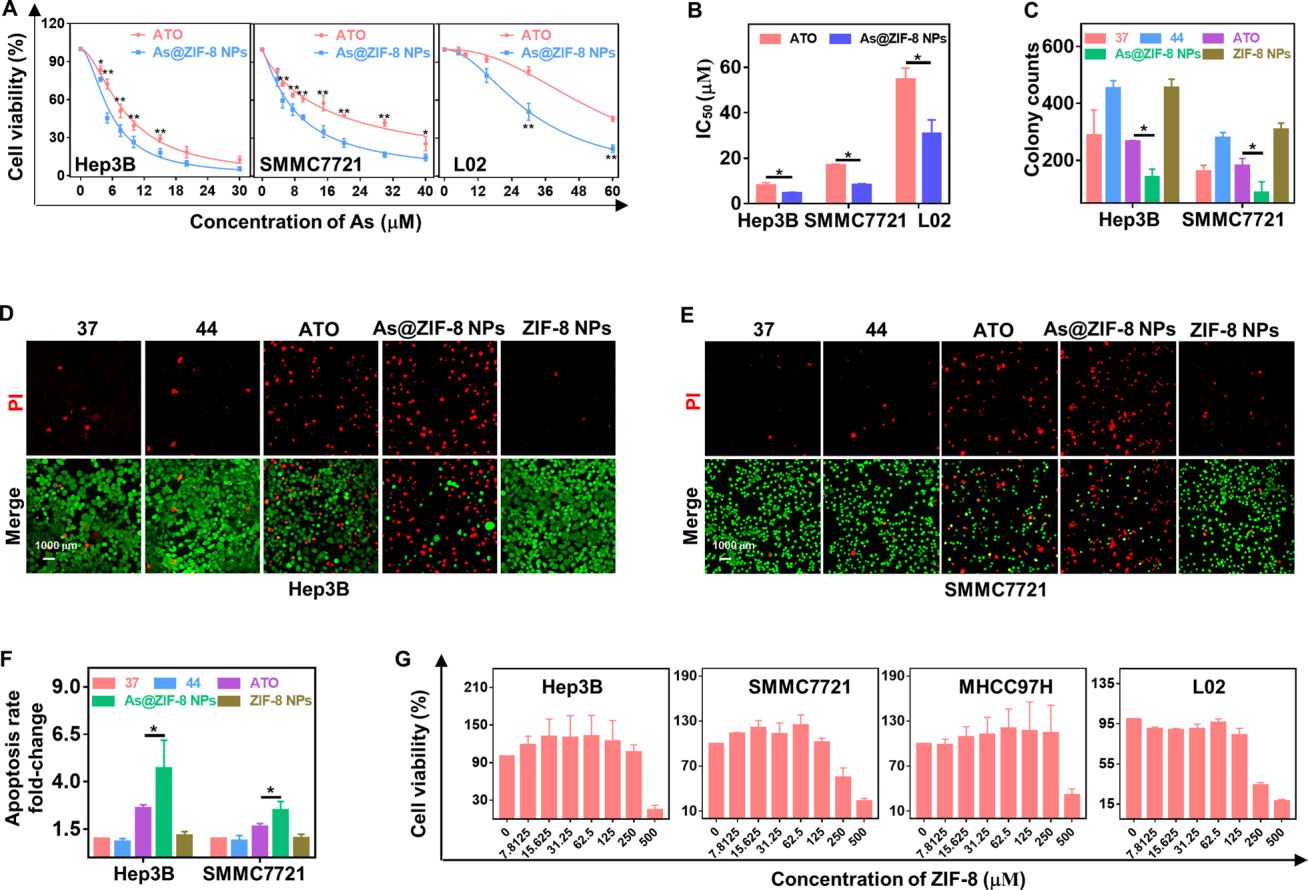
As@ZIF-8 NPs inhibited the proliferation and promoted the apoptosis of sublethally heated cells in vitro. **A** Viability of Hep3B, SMMC7721, and L02 cells after incubation with the indicated concentrations of free ATO or As@ZIF-8 NPs for 48 h (n = 3). **B** IC_50_ of Hep3B, SMMC7721, and L02 cells after treatment with ATO or As@ZIF-8 NPs (n = 3). **C** Quantitative analysis chart of colony formation of sublethally heated Hep3B and SMMC7721 cells after incubation with free ATO, As@ZIF-8 NPs or ZIF-8 NPs for 24 h (n = 3). **D**, **E** Living/dead cell double staining of sublethally heated Hep3B (**D**) and SMMC7721 cells (**E**) after the indicated treatment. Scale bar, 1000 µm. **F** Quantitative analysis chart of apoptosis rates of sublethally heated Hep3B and SMMC7721 cells after incubation with free ATO, As@ZIF-8 NPs or ZIF-8 NPs (n = 3). **G** Viability of Hep3B, SMMC7721, MHCC97H, and L02 cells after incubation with the indicated concentrations of ZIF-8 NPs for 48 h (n = 3). (*P < 0.05; **P < 0.01)

First, FITC-labeled ZIF-8 NPs were employed to trace the process of cellular uptake by HCC cells. A portion of the FITC@ZIF-8 NPs was internalized by Hep3B cells after approximately 40 min of incubation, and these NPs were evenly distributed in cells within 8 h (Additional file 1: Fig. S7). Furthermore, at concentrations up to 150 μM, the ZIF-8 nanocarrier induced negligible toxicity in three HCC cell lines and L02 hepatocytes (Fig. 3G). In addition, hepatocytes and HCC cells were incubated for 48 h with ATO or As@ZIF-8 NPs at a series of As concentrations, and the half-maximal inhibitory concentrations (IC_50_) were calculated. The results revealed that As@ZIF-8 NPs showed a greater effect than ATO in suppressing HCC cell survival (Fig. 3A). The IC_50_ values of As@ZIF-8 NPs in Hep3B and SMCC7721 cells were 4.58 ± 0.25 μΜ and 8.21 ± 0.31 μΜ, respectively, whereas those of ATO were 8.05 ± 0.65 μΜ and 16.80 ± 0.31 μΜ, respectively (Fig. 3B). Notably, the IC_50_ value of As@ZIF-8 NPs in L02 hepatocytes was 54.52 ± 3.00 μΜ (Fig. 3B). This difference in IC_50_ values indicated that As@ZIF-8 NPs induced markedly lower cytotoxicity in normal hepatocytes. Consistently, a colony formation assay showed that As@ZIF-8 NPs induced more obvious inhibition of the colony formation of sublethally heated Hep3B and SMMC7721 cells than that obtained with ATO (Additional file 1: Fig. S8, Fig. 3C). Furthermore, double staining of living dead cells demonstrated that As@ZIF-8 NPs exhibited more efficacy in killing HCC cells than ATO (Fig. 3D, E, Additional file 1: Fig. S9). The cell apoptosis induced by As@ZIF-8 NPs was also analyzed by flow cytometry. The incubation of sublethally heated Hep3B and SMMC7721 cells with the As@ZIF-8 NPs for 36 h induced apoptosis at 2.81 ± 0.30- and 2.56 ± 0.23-fold higher rates compared with the rates obtained with ATO, respectively (Additional file 1: Fig. S10, Fig. 3F).

In all the aforementioned experiments, the ZIF-8 nanocarrier showed a negligible effect. These results collectively suggested that As@ZIF-8 NPs showed satisfying therapeutic efficacy against sublethally heated HCC cells in vitro.

### As@ZIF-8 NPs inhibited the migration, invasion and EMT of sublethally heated HCC cells in vitro

**Fig. 4:**
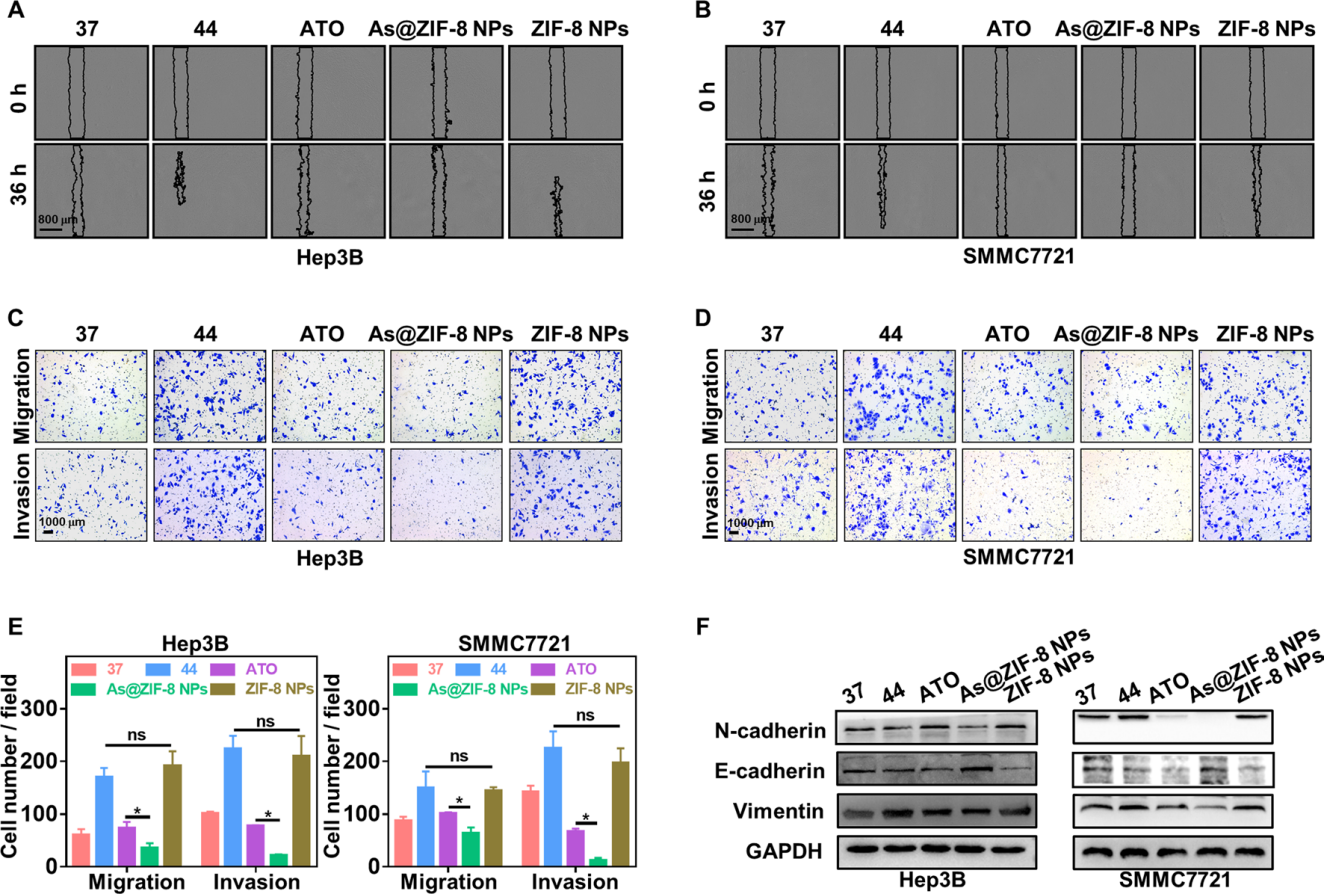
As@ZIF-8 NPs inhibited the invasion, migration and EMT of sublethally heated cells in vitro. **A**, **B** Wound healing assay of sublethally heated Hep3B (**A**) and SMMC7721 cells (**B**) after incubation with free ATO, As@ZIF-8 NPs and ZIF-8 NPs for 24 h. Scale bar, 800 µm. **C**–**E** Representative images of the migration and invasion of sublethally heated Hep3B (**C**) and SMMC7721 cells (**D**) after the indicated treatment and the related quantitative analysis chart (**E**). Scale bar, 1000 µm. **F** Protein expression of EMT markers N-cadherin, vimentin and E-cadherin in Hep3B and SMMC7721 cells after the indicated treatment. (ns, no statistical difference; *P < 0.05)

Since residual tumor cells are characterized by enhanced metastatic ability, we delineated the effect of As@ZIF-8 NPs on the invasion and migration of sublethally heated HCC cells. First, the healing rate of a wound monolayer at the indicated time points revealed that As@ZIF-8 NPs more efficiently inhibited the migration of sublethally heated HCC cells than ATO (Fig. 4A, B, Additional file 1: Fig. S11). Moreover, the results of the transwell migration assay indicated that As@ZIF-8 NPs inhibited the migration of sublethally heated Hep3B and SMMC7721 cells by 79.06 ± 1.72% and 57.22 ± 1.02%, respectively, whereas ATO decreased their migration rate by 57.34 ± 1.63% and 30.35 ± 8.39%, respectively (Fig. 4C–E). The transwell invasion assay also revealed that As@ZIF-8 NPs potently reduced the invasive ability of sublethally heated Hep3B and SMMC7721 cells by 90.27 ± 0.25% and 94.79 ± 0.87%, respectively, whereas the relative inhibitory rates of ATO were 64.96 ± 2.08% and 69.89 ± 2.02%, respectively (Fig. 4C–E). In addition, a western blot analysis showed that the upregulation of mesenchymal markers N-cadherin and vimentin upon cell exposure to sublethal heat was down-regulated more significantly by As@ZIF-8 NPs than by ATO. The epithelial marker E-cadherin was upregulated most obviously after As@ZIF-8 NPs treatment (Fig. 4F). However, sublethally heated Hep3B and SMMC7721 cells treated with ZIF-8 NPs failed to show these types of apparent changes. These observations indicated that As@ZIF-8 NPs were more effective in suppressing the migration- and invasion-like changes and EMT of sublethally heated tumor cells.

### Enhanced enrichment of ICG@ZIF-8/PEG in residual tumors after IRFA

**Fig. 5:**
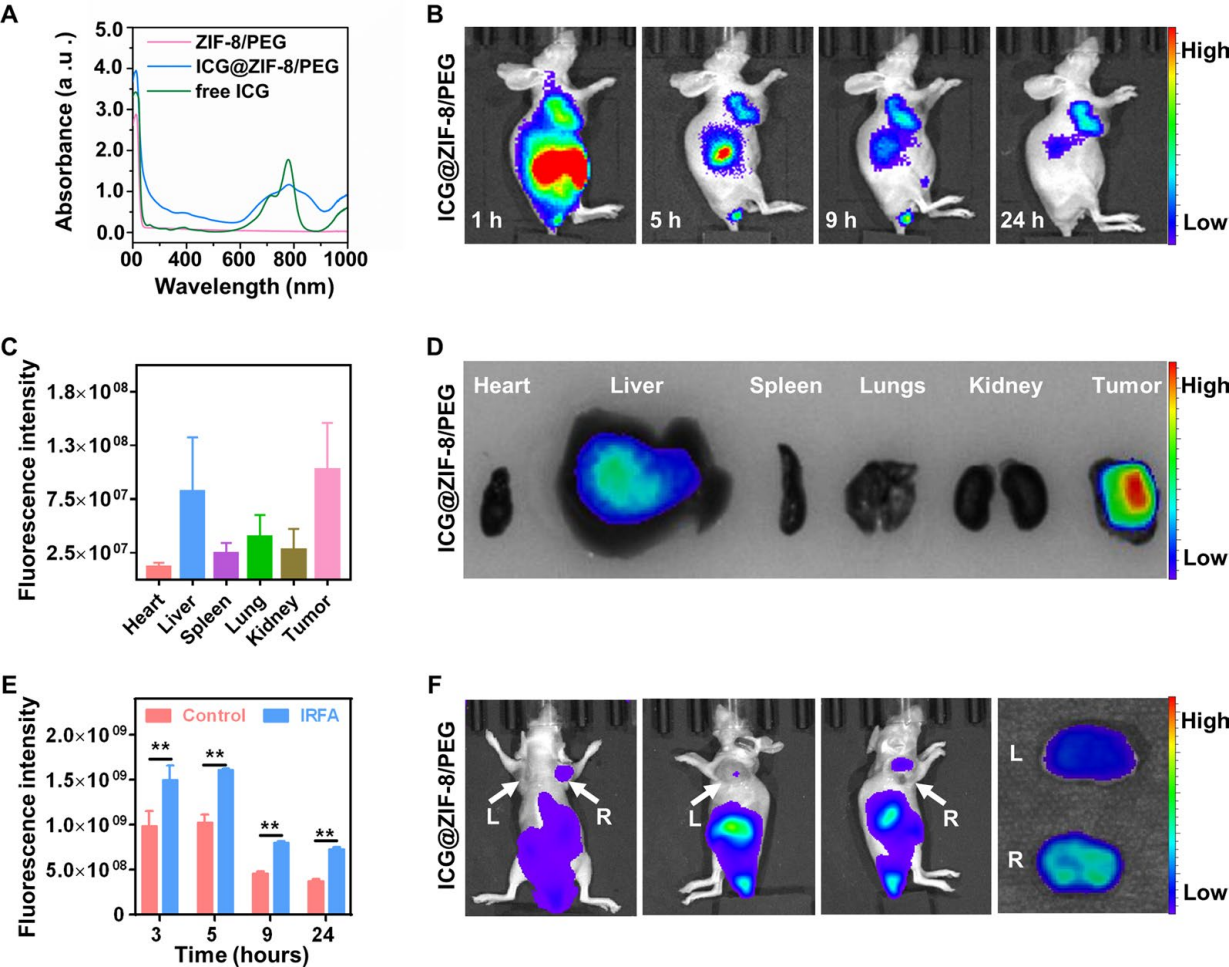
Augmented enrichment of ICG@ZIF-8 NPs in residual tumors after IRFA. **A** UV–vis absorption absorbance spectra of ZIF-8 NPs, free ICG, and ICG@ZIF-8 NPs. **B** In vivo fluorescence imaging of ICG@ZIF-8 NPs in tumor-bearing nude mice. **C**, **D** Fluorescence imaging of dissected organs and tumors (**D**) and the related quantitative analysis chart (n = 4) (**C**). **E**, **F** In vivo enrichment of ICG@ZIF-8 NPs in tumors with or without IRFA (**F**) and the related quantitative analysis chart (n = 3) (**E**). R, IRFA. L, sham RFA (**P < 0.01)

To explore the in vivo distribution of NPs in HCC tumor-bearing nude mice, we successfully synthesized ICG-labeled ZIF-8 NPs (Additional file 1: Fig. S12). The UV–vis absorption spectra of ICG@ZIF-8 NPs exhibited the characteristic ICG absorption band at 780 nm (Fig. 5A), which illustrated the successful loading of ICG on ZIF-8 NPs. As shown in Fig. 5B, a strong fluorescence signal appeared in the tumor region 1 h after injection, and this signal then gradually decreased over time and lasted for at least 24 h. Furthermore, the ex vivo images taken 24 h after injection showed that ICG@ZIF-8 NPs mainly accumulated in the liver and tumor (Fig. 5C, D). This outcome vividly showed that these NPs were enriched in the tumor site through the EPR effect. Through ultrasound imaging (Fig. 1F) and CD34 immunohistochemistry staining (Additional file 1: Fig. S5), we previously confirmed that IRFA can promote angiogenesis in residual cancer. Extensive angiogenesis is an important pathophysiological factor associated with the EPR effect. Based on these findings, we investigated whether angiogenesis induced by IRFA led to additional increases in the accumulation of NPs. Unilateral IRFA was performed in mice with bilateral upper limb tumors, and ICG@ZIF-8 NPs were injected via the caudal vein 7 days after IRFA (Additional file 1: Fig. S13). Interestingly, throughout the observation period, we found that the residual tumors after IRFA emitted a more intense fluorescence signal than the tumors after sham IRFA (Fig. 5E, F). Similarly, the ex vivo fluorescence signal of the dissected tumors displayed consistent results (Fig. 5F). These results suggested that NPs can further be accumulated in the residual tumor after IRFA due to the augmented EPR effect, which may tremendously improve the therapeutic efficacy of As@ZIF-8 NPs against residual tumors.

### As@ZIF-8 NPs inhibited residual tumor growth and metastasis in vivo

**Fig. 6:**
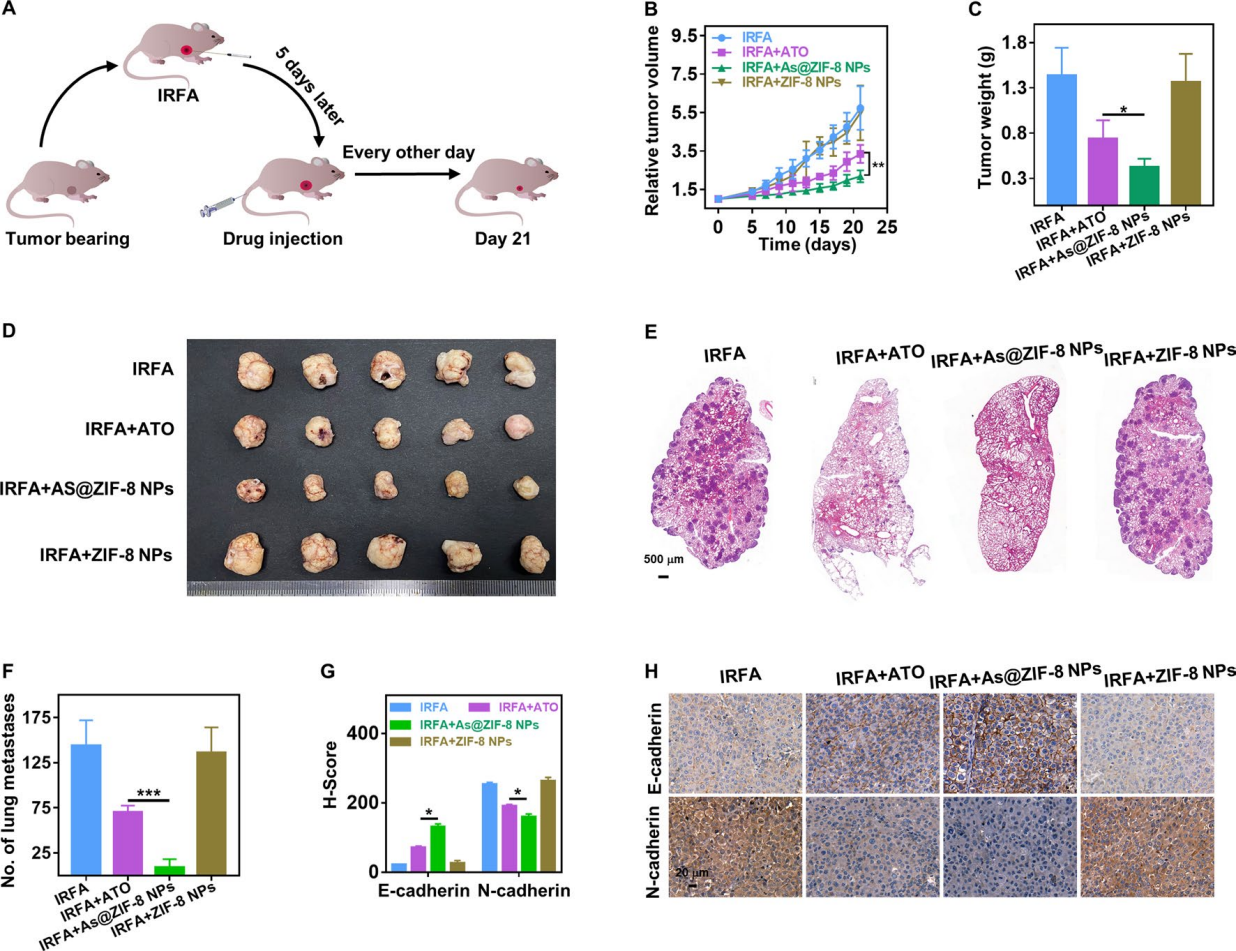
As@ZIF-8 NPs inhibited the growth and metastasis of residual tumors in vivo. **A** Schematic diagram of subcutaneous tumor treatment. **B** Tumor growth curve of the mice after different treatments (n = 5). **C** Tumor weight obtained from the mice receiving different treatments (n = 5). **D** Photographs of excised tumors 21 days after different treatments were administered. **E**, **F** HE staining (**E**) of lung metastasis area after different treatments and quantification (n = 5) (**F**). Scale bar, 500 µm. **G**, **H** Immunohistochemical staining (**H**) of N-cadherin and E-cadherin in tumor tissues 21 days after different treatments were administered and quantification of the H-score (n = 5) (**G**). H-score, histochemistry score. Scale bar, 20 µm (*P < 0.05; **P < 0.01; ***P < 0.001)

Encouraged by the satisfactory efficacy of As@ZIF-8 NPs in vitro and their enhanced enrichment in residual tumors after IRFA, we sought to evaluate whether these effects translate into therapeutic results in nude mice bearing HCC tumors. To perform this analysis, we randomly assigned mice into four groups (5 mice per group) once the tumors grew to 200–400 mm^3^ and then subjected the mice to IRFA. The mice were then intravenously injected with free ATO, As@ZIF-8 NPs or ZIF-8 NPs every other day from day 5 to day 21 after IRFA (Fig. 6A). The tumor volumes were monitored and the tumor growth curve is plotted in Fig. 6B. The As@ZIF-8 NPs group showed the most obvious inhibition of residual tumor growth, while the ZIF-8 nanocarrier showed a neglected effect. Notably, tumor growth was also partially delayed by ATO, but the antitumor effect of ATO was lower than that of the As@ZIF-8 NPs. The images of dissected tumor tissue on day 21 indicated that the tumor size of the As@ZIF-8 NPs group was smaller than that of the other groups, whereas the tumor size of the free ATO group was smaller than that of the control group but much larger than that of the As@ZIF-8 NPs group (Fig. 6D). In addition, the antitumor rate of the As@ZIF-8 NPs group was 81.55 ± 3.14%, which was higher than that of the free ATO (60.83 ± 2.93%) (Fig. 6C, Additional file 1: Fig. S14).

**Fig. 7:**
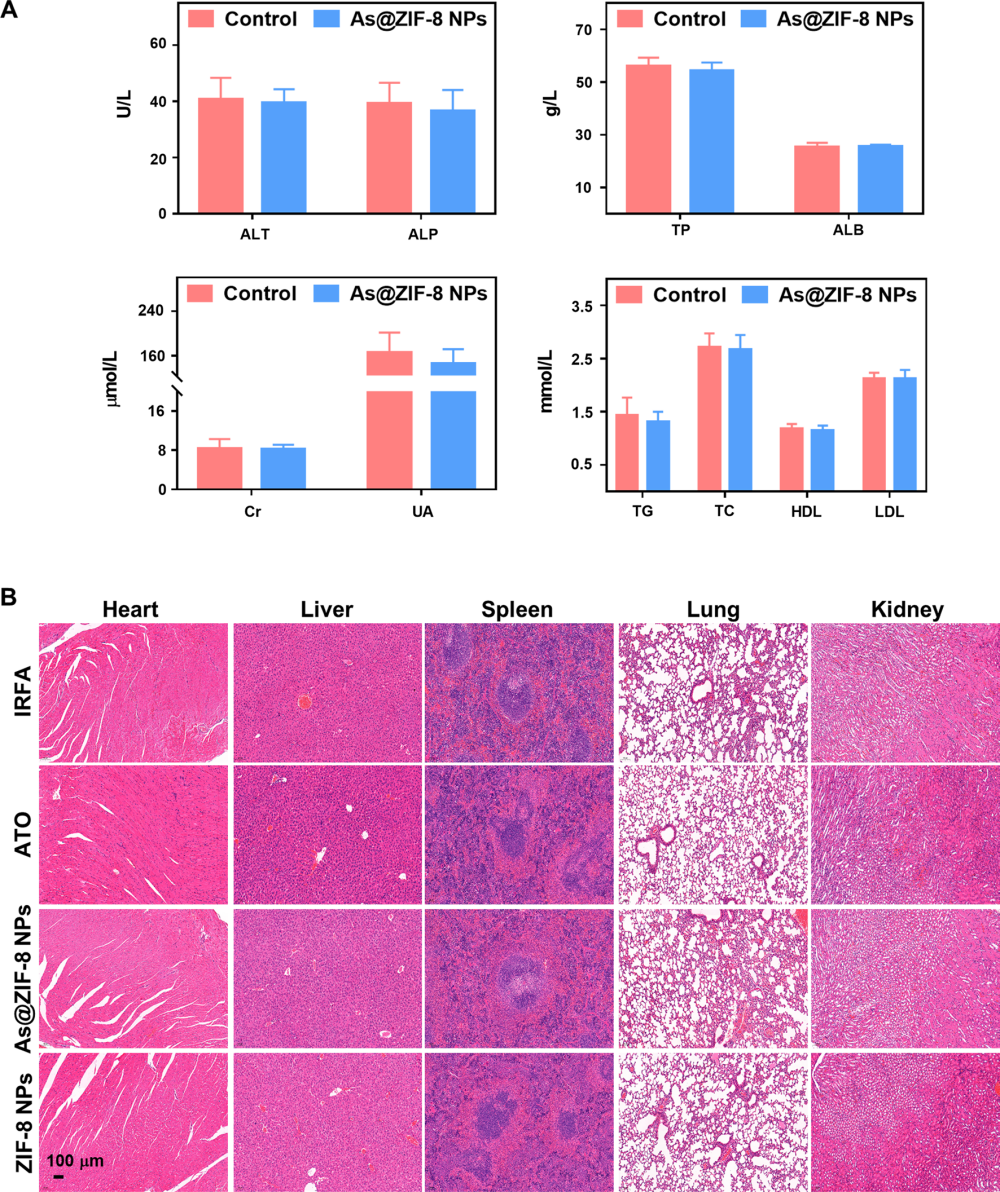
Biosafety assessment of the As@ZIF-8 NPs. **A** Blood analysis of alanine aminotransferase (ALT), alkaline phosphatase (ALP), total protein (TP), albumin (ALB), creatinine (Cr), uric acid (UA), triglyceride (TG), total cholesterol (TC), high density lipoprotein (HDL), and low density lipoprotein (LDL) levels after treatment with free ATO or As@ZIF-8 NPs. **B** Photographs showing HE staining of tissue sections in important organs, namely, the liver, lung, heart, spleen and kidney, of the mice after treatment with free ATO, As@ZIF-8 NPs, or ZIF-8 NPs. Scale bar, 100 µm

In addition, all the mice maintained negligible weight fluctuations during treatment, which indicated that the As@ZIF-8 NPs induced few side effects (Additional file 1: Fig. S15). Hematoxylin and eosin (HE) staining revealed no obvious histopathological changes in major organs (Fig. 7B). Blood tests also indicated that As@ZIF-8 NPs were biocompatible (Fig. 7A).

To investigate the therapeutic efficacy of As@ZIF-8 NPs on metastatic inhibition of HCC cells in vivo, sublethally heated cells were injected into mice to establish a lung metastasis model. An analysis of these mice showed that As@ZIF-8 NPs reduced the metastasis of residual tumors in vivo to a greater extent than ATO (Fig. 6E, F). Consistently, an immunohistochemical analysis of subcutaneous tumors in all the groups showed that most notable suppression of the expression of N-cadherin and the greatest upregulation of the expression of E-cadherin were obtained with the As@ZIF-8 NPs (Fig. 6G, H). These results suggested that application of As@ZIF-8 NPs may be a promising strategy for residual tumor therapy while providing good biocompatibility.

## Discussion

The rapid progression of residual tumor after IRFA remains a primary clinical challenge. IRFA is attributed to several risk factors, such as the heat sink effect of major vessels adjacent to a tumor [51], a irregular tumor morphology or an excessive volume of tumors [52], and the tumor location in close proximity to important organs or tissues [53]. To achieve technical success, the ablation zone must sufficiently include the tumor and a safety margin encompassing at least 5 mm of normal tissue [54]. However, the precise ablation boundary cannot always be readily discerned using the currently applied clinical imaging techniques. This lack of clarity also leads to the increased occurrence of residual tumors after RFA. Moreover, sublethal IRFA temperatures can endow residual tumors with an aggressive phenotype, which makes them intractable to treatment.

EMT is activated by IRFA and is critical for the invasion and dissemination of residual HCC tumor cells [3, 10]. Several studies have demonstrated that IRFA increases the expression of EMT-inducing transcription factors (EMT-TFs), such as snail, slug and twist1, in remnant cancer cells. These EMT-TFs orchestrate the EMT process, which includes the repression of genes associated with the epithelial state, the upregulation of mesenchymal state related genes, the inhibition of genes associated with lateral cell–cell junctions and apical–basal polarity, and the induction of matrix metalloproteinase expression [55]. The importance of IRFA in residual cancer was also demonstrated by downregulated expression of E-cadherin and upregulated expression of N-cadherin and vimentin in the sublethally heated cells analyzed in the present study (Fig. 1D, H). Notably, cells display front–rear polarity, and the interactions of cells with other cells and the extracellular matrix involve remodeling, which may drive residual HCC cells into a motile and disseminate state [55]. This remodeling may partially explain the superior migration and invasion potential of the sublethally heated cells in our results (Fig. 1C, G). These malignant translations eventually confer residual HCC cells with strong metastatic potential and resistance to chemotherapy, which are formidable challenges in clinical practice [3, 5].

Enhanced angiogenesis is another crucial factor that leads to the malignant progression of residual HCC after IRFA. Previous studies have shown that IRFA induces hypoxia in tumor tissue in the transition zone. Subsequently, proangiogenic factors such as hypoxia inducible factor-1α and vascular endothelial growth factor (VEGF) are generated to drive neovascularization around the necrotic lesion after IRFA [9]. Consistent with previous research, we observed that IRFA promoted HUVEC tube formation in vitro (Fig. 1E). Furthermore, our study clearly validated the finding of an increase in the percentage of blood vessels in residual cancer after IRFA in vivo (Fig. 1G). As a result of this angiogenesis, increases in supplied nutrients and abundant oxygen rapidly accelerate the growth of residual tumors. In a particularly deleterious outcome, newly formed tumor vessels exhibit immature morphological features and disorganized vascular networks, which may further facilitate the intravasation of residual tumor cells to colonize distant destinations together with the effects of EMT [55, 56]. These factors potently promote the progression of residual tumors and seriously impede the effects of treatment.

Although great efforts have been made to eliminate residual tumors after RFA, the consequences of IRFA remain obstacles. Therefore, we sought to explore a more effective solution based on the intrinsic characteristics of residual tumors. Solid tumors are characterized by highly selective permeability and the retention of macromolecular substances and lipid particles, and collectively, these outcomes are referred to as the EPR effect [56, 57]. EPR is spontaneously derived through particular anatomical and pathophysiological characteristics of solid tumors, such as an immature and abnormal vascular structure, an impaired lymphatic function and abundant levels of vascular mediators, including bradykinin, nitric oxide, prostaglandins, proinflammatory cytokines, and interleukins [56]. Recently, several tactics have been applied to augment the EPR effect, and one of these strategies involves enhancing angiogenesis by administering angiogenic drugs [57]. Inspired by the strategy of vascular promotion, the induction of extensive angiogenesis after IRFA seems to be a suitable approach for promoting the EPR effect and nanodrug enrichment. To test our hypothesis, a unilateral IRFA tumor model was constructed, and we confirmed that ICG@ZIF-8 NPs were further enriched in residual cancer after IRFA (Fig. 5E, F). In addition to increasing angiogenesis, several other subtle IRFA-induced changes in the tumor microenvironment may facilitate the enrichment of nanocarriers in tumors. First, previous studies have shown that IRFA can increase the infiltration of leukocytes, which produce nitric oxide and thus subsequently increase vascular permeability [2, 4]. Second, some proinflammatory cytokines (tumor necrosis factor-α and interleukins) and matrix metalloproteinases can be activated after IRFA and promote vascular permeability through direct or indirect pathways [2]. Third, the hyperthermia caused by RFA may damage the tumor lymphatic vessels, and this eventually results in the prolonged retention of NPs at tumor sites. Considering the results of these analyses, we propose that residual cancer after IRFA may provide a natural anatomical and pathophysiological basis for enhancing the EPR effect.

Encouraged by the augmented EPR effect observed in residual tumors post IRFA, we prepared ATO-loaded ZIF-8 NPs and explored their biosafety and therapeutic outcomes. First, the As@ZIF-8 NPs exhibited excellent biocompatibility (Fig. 7A, B), and this characteristic can be primarily attributed to their satisfactory stability in neutral environments and their biodegradability under acidic conditions (Fig. 2M). This satisfactory stability ensures minimal drug release in the neutral environment of blood and minimizes side effects in vivo to a greater extent than conventional chemotherapy. The superior biodegradability of As@ZIF-8 NPs and their harmless degradation products may indicate that these NPs pose few potential safety hazards and a greater likelihood of clinical transformation. As a treatment, As@ZIF-8 NPs exhibited substantially increased therapeutic efficacy in residual tumor growth suppression, metastasis inhibition and EMT reversal compared with the effects of ATO in vitro and in vivo (Figs. 3, 4, 6). The reasons for these outcomes can be deduced from our review and several findings. First, sublethal heat leads to excessive angiogenesis and a self-augmented EPR effect, which resluts in increased drug accumulation in residual tumor sites. Second, solid tumors are characterized by acidic microenvironments. As@ZIF-8 NPs showed a higher level of arsenic ion release at pH 5.5, indicating that high concentrations of arsenic can be released in the acidic tumor microenvironment and tumor lysosomes, which is greatly conducive to the arsenic-mediated induction of apoptosis and inhibition of the proliferation, metastasis and EMT of residual tumor cells. Third, the nanoparticle size and PEG modification may prolong the circulation life of As@ZIF-8 NPs, which may result in better bioavailability and stronger therapeutic effects in vivo. Fourth, it has been reported that ZIF-8 can positively affect the antitumor effect of chemotherapeutic drugs by altering the drug delivery pathway in the cells [41, 58]. In more detail, ZIF-8 can more rapidly distribute drugs to whole cells and not just to the nucleus and destroy the function of mitochondria, which results in more rapid cell death than that observed with free drugs. Previous studies have also shown that the zinc level in hepatoma cells is significantly lower than that in normal liver cells, which is related to the carcinogenesis and progression of HCC [59]. Zinc released by ZIF-8 degradation may promote the sensitivity of cancer cells to chemotherapeutic drugs and inhibit the development of HCC [60, 61]. These may partly explain why the As@ZIF-8 NPs group showed lower IC_50_ values and less invasion and migration of residual cells than the ATO group.

In this study, several concerns warrant attention and further study. First, the augmented EPR effect induced by IRFA is a dynamic pathophysiological process. At the early post-RFA stage, the blood supply and vascular density in residual cancer are decreased, and the EPR effect may not have been obvious. Due to the formation of a hypoxic microenvironment in remnant tumor, angiogenesis is promoted evenly throughout the residual tumor, which gradually augments the EPR effect. However, with the excessive growth of residual tumors, some blood vessels in tumors collapse, and perfusing blood is unevenly concentrated, which may lead to the inhomogeneous enrichment of nanodrugs. Therefore, it is important to determine the proper time point for combining nanodrugs with the treatment of residual HCC after IRFA. Second, many pharmacological strategies can enhance the EPR effect, and these strategies include improving vascular permeability with inflammatory cytokines, achieving vessel normalization via anti-VEGF(R) agents and enhancing angiogenesis through the use of angiogenic drugs [57]. Hence, the nanoparticle codelivery of vascular mediators with ATO may further augment the EPR effect and increase its effectiveness. Third, RFA induces cell necrosis and the release of cellular components, which may lead to an inflammatory response and thus to the infiltration of many innate immune cells and adaptive immune cells into residual cancer sites [4]. Therefore, the coating As@ZIF-8 biomimetic NPs with cell membrane to target the immune microenvironment may improve their effectiveness in inhibiting residual tumor growth. Moreover, it is also possible to reduce drug release in the blood circulation. Fourth, synthesis of As@ZIF-8/PEG based on electrostatic adsorption may inevitably lead to drug leakage; therefore, the in situ loading of arsenic into the pores of the framework during crystal growth may help reduce As release under neutral conditions.

## Conclusion

In summary, we confirmed that IRFA can markedly promote the malignant transition of residual HCC tumors. Hence, we prepared arsenic loaded ZIF-8 NPs to inhibit residual tumor progression. Importantly, our results provided the first demonstration that extensive angiogenesis after IRFA augmentd the EPR effect and enhanced ZIF-8 nanocarrier enrichment in residual tumors. Based on this targeted accumulation, the As@ZIF-8 NPs exhibit strongly enhanced therapeutic efficacy in inhibiting residual tumor growth, metastasis and EMT compared with ATO. Thus, this work provides a proof of concept for the treatment of residual HCC after IRFA.

## Materials and methods

### Materials

Phosphate-buffered saline (PBS), Trypsin/EDTA solution, Dulbecco’s modified Eagle’s medium (DMEM), fetal bovine serum (FBS), and penicillin/streptomycin (P/S) double antibody were purchased from Gibco. Accutase® solution and NaAsO_2_ were purchased from Sigma-Aldrich. Endothelial cell medium (ECM) was purchased from ScienCell Research Laboratories. Primary antibodies (anti-vimentin and anti-E-cadherin) were purchased from Cell Signaling Technologies. Additional primary antibodies (anti-CD34, anti-N-cadherin) were purchased from Abcam. A Cell Counting Kit-8 (CCK-8) was purchased from DOJINDO. Zn(NO_3_)_2_·6H_2_O, 2-methylimidazole, polyethylene glycol (2000), indocyanine green (ICG) and fluorescein isothiocyanate (FITC) were purchased from Aladdin. Methanol was purchased from Guangzhou Chemical Reagent Factory. An Annexin V-FITC/PI apoptosis detection kit was purchased from BD Biosciences. A calcein-AM/PI double staining kit was purchased from Solarbio, Beijing.

### Preparation of As@ZIF-8/PEG

Firstly, 150 mg of zinc nitrate hexahydrate was dispersed in 7 mL of methanol and labeled solution A. Then, 7 mL of a dimethyl imidazole methanol solution (50 mg/mL) was added dropwise into solution A and mixed for 15 min. A white product was acquired by centrifugation, washed twice with methanol, redistributed into 2 mL of ultrapure water and labeled solution B. Then, 1 mL of a NaAsO_2_ solution (60 mg/mL) was mixed with solution B for 15 min, and 30 μL of COOH–PEG–COOH (40 mg/mL) was subsequently added to obtain As@ZIF-8/PEG. ZIF-8 NPs were prepared following the same method without the addition of NaAsO_2_.

### Characterization

The morphology and elemental components of ZIF-8 and the As@ZIF-8 NPs were verified by transmission electron microscopy (TEM) and energy dispersive spectroscopy (EDS) mapping (TecnaiG2, FEI, USA). The crystalline structure was determined by X-ray diffraction (XRD; D-MAX 2200 VPC diffractometer, Rigaku, Japan). The functional groups were observed by Fourier transform infrared spectrometry (FTIR; EQUINOX55 spectrometer, Bruker, Germany). The chemical composition and chemical oxidation states were determined by X-ray photoelectron spectrometry (XPS, ESCALAB 250, Thermo Fisher, USA). UV–vis absorption spectra were obtained with an ultraviolet visible spectrophotometer (LAMBDA 365, PerkinElmer, USA). The zeta potential was measured by a laser nanometer (Zetasizer Nano ZSE, Malvern, U.K.). Quantitative analysis of the elemental components was detected by inductively coupled plasma-optical emission spectrometry (ICP-OES; Varian 700, Varian, USA). Fluorescence imaging of cells was recorded by confocal laser scanning microscopy (CLSM; Zeiss 880, Zeiss, Germany).

### In vitro drug release

To explore the release pattern of the nanomedicine in vitro, 20 mg As@ZIF-8/PEG was suspended in 20.0 mL of PBS (pH = 7.5 or 5.5) at room temperature. After agitation for a predetermined time, 1 mL of liquid was poured into a centrifuge tube and centrifuged for 15 min, and the supernatant was then collected. Then, 1 mL of fresh PBS with the corresponding pH was added to the buffer solution. The amount of released arsenic was verified by ICP-OES.

### Cell lines and cell culture

Hepatoma cell lines (Hep3B, SMMC7721, HCC-LM3 and MHCC97H cells), L02 hepatocytes and human umbilical vein endothelial cells (HUVECs) had been previously acquired from the Cell Bank of the Chinese Academy of Sciences (Shanghai, China). The HCC cell lines and L02 cells were grown in DMEM containing 10% FBS and 1% P/S double antibody. The HUVECs were grown in ECM containing 5% FBS, 1% P/S and 1% endothelial cell growth supplement. All the cell lines were incubated in a humidified atmosphere of 5% CO_2_ at 37 °C.

### Sublethally heated cell model

A sublethally heated cell model was established according to the literature [5]. HCC cells were seeded into plates. After 24 h, the culture medium was replaced with preheated medium. Then, the plates were immediately sealed with parafilm and subsequently submerged in preheated water baths (37, 42, 44, 46, 48 °C). After heating for 15 min, the plates were transferred to an incubator maintained at 37 °C with 5% CO_2_ for 24 h in preparation for use in subsequent experiments.

### Colony forming test

Cells were seeded at 2 × 10^3^ cells per well into six-well plates. After 11 days of cultivation, cell clones were fixed in cold methanol and stained with crystal violet (Solarbio, Beijing, China), and the number of cell clones was counted.

### Cell viability assay

A CCK-8 (DOJINDO, Kumamoto, Japan) was adopted to detect cell viability. Briefly, cells were seeded into 96-well plates at 5 × 10^3^ cells per well for 24 h, incubated with the indicated concentrations of ATO, As@ZIF-8 NPs or ZIF-8 NPs for 48 h and cultured with medium containing 10% CCK-8 solution for 2 h. A multimode reader (Synergy HTX, Bio-Rad, USA) was used to record the absorbance value at 450 nm, and then, cell viability was calculated.

### Cellular uptake experiment

Hep3B cells were seeded in confocal culture dishes at 8 × 10^4^ cells per dish for 24 h and incubated with FITC@ZIF-8 NPs for 40 min, 4 h or 8 h. Then, the cells were washed twice with PBS, fixed in cold methanol for 25 min, and incubated with DAPI (Solarbio, Beijing, China) at room temperature for 5 min. The green fluorescence signal was captured by CLSM at an excitation wavelength of 488 nm.

### Migration and invasion assays

Transwell chambers (Corning Inc.) were used to carry out migration assays. Then, 100 μL of serum-free DMEM containing 20,000 cells was placed into the upper chamber, and 800 μL of DMEM containing 10% FBS was added to the lower chamber. After 36 h, the cells that migrated to the lower chamber were fixed in cold methanol for 15 min, stained with crystal violet for 1 h, washed three times with PBS, and photographed at 100× magnification with a microscope. For the cell invasion assay, most of the steps were the same as those in the cell migration assay, except that in the invasion assay, the Transwell membrane was precoated with 50 μL of Matrigel (0.3 mg/mL; Corning, USA) before the cells were plated.

### Wound healing assay

Sublethally heated cells were treated with drugs (ATO, As@ZIF-8 NPs or ZIF-8 NPs) for 24 h, and then living cells were seeded into six-well plates at 6 × 10^5^ cells per well. Twenty-four hours later, several scratches were made with the tip of a 200-μL pipette. The plates were transferred to an automatic cell imaging system (IncuCyteS3, Essen Bioscience, USA) maintained at 37 °C and photographed every 12 h for 36 h. The migration area was quantified with ImageJ analysis software (ImageJ, NIH, USA).

### Tube formation assay

HUVECs were heated for 15 min at 44 °C and cocultured for 24 h with supernatant from sublethally heated HCC cells added at a ratio of 1:1. Then, 100 µL of the HUVEC suspension (1.2 × 10^5^/mL) was added to a 96-well culture plate precoated with Matrigel (10 mg/mL; Corning, USA). The plate was incubated in an automatic cell imaging system and photographed every 30 min for 12 h. The total branching length, nodes and meshes were quantified with ImageJ analysis software.

### Cell apoptosis analysis and double staining with a living/dead cell assay kit

Sublethally heated cells were treated with drugs (ATO, As@ZIF-8 NPs or ZIF-8 NPs) for 36 h and harvested and stained with Annexin V-FITC and PI solution for the evaluation of apoptosis. The apoptosis rate was determined on the basis of the flow cytometry results (CytoFLEX LX, Beckman Coulter, USA), which were analyzed by CytExpert 2.0 software (Beckman Coulter, USA). Similarly, drug-treated HCC cells were stained with calcein-AM and PI solution to assess the number of living and dead cells. The fluorescence signals were observed by CLSM at excitation wavelengths of 488 nm for calcein-AM (living cells) and 555 nm for PI (dead cells).

### Western blot analysis

Protein samples were obtained by lysing cells on ice with RIPA buffer containing 1% protease inhibitor (Thermo Fisher, Massachusetts, USA), segregated by SDS-PAGE and transferred to polyvinylidene difluoride (PVDF) western blot membranes (Sigma-Aldrich, Missouri, USA). Then, the membranes were blocked for 1.5 h, incubated with primary antibodies (anti-GAPDH, anti-E-cadherin, anti-vimentin, and anti-N-cadherin) overnight at 4 °C and then incubated with horseradish peroxidase (HRP)-conjugated secondary antibody (ZSGB-BIO, Beijing, China) for 1 h. A gel chemiluminescence imager (ChemiDoc/XPS+, Bio-Rad, USA) was used to detect the proteins on the membranes.

### IRFA subcutaneous tumor model and lung metastasis model

HCC-LM3 cells were subcutaneously inoculated into the lower flanks of BALB/c nude mice to induce tumor growth. When the tumors grew to 200–400 mm^3^, the mice were anesthetized and positioned on a VIVA grounding pad. The RF electrode was inserted into the tumor and positioned through one-third of the length of the tumor under the guidance of ultrasound. IRFA was performed with a power output of 5 W and a duration of 20 s; sham IRFA was performed with no power output. The bioluminescence of the tumors was used to confirm that the models had been established successfully. To establish a lung metastasis model, 8 × 10^5^ sublethally heated MHCC97H cells in 200 μL of PBS were intravenously injected into each model mouse.

### Effect of antitumor growth and antitumor metastasis in vivo

Five days after model establishment, ATO, As@ZIF-8 NPs and ZIF-8 NPs were intravenously injected into mice every other day. The dose of ATO and As@ZIF-8 NPs was 1 mg As/kg, and the dose of ZIF-8 NPs was the same as that of As@ZIF-8 NPs. To evaluate the effect of inhibiting tumor growth, tumor volume was recorded with the following formula: volume = (width^2^ × length)/2. Twenty-one days after IRFA, the heart, tumor, liver, kidney, spleen and lungs were resected for pathological examination, and blood was collected for biochemical analysis. To evaluate the effect of inhibiting lung metastasis, lung tissue was extracted for HE staining 22 days after cell injection, and the metastases on the largest HE-stained section were counted.

### High-frequency ultrasound vascular imaging

A Vevo 3100 Imaging System (VisualSonics, CA) was used to evaluate the distribution of tumor vessels before RFA and 7 days after RFA. Briefly, mice anesthetized upon inhaling 4% isoflurane were positioned on a heated monitoring table. Power Doppler mode was used to display tumor vessels, and 3D video data were recorded. All data were analyzed by Vevo LAB 3.1.1 software.

### In vivo fluorescence imaging

When tumors had grown to 300–400 mm^3^, ICG@ZIF-8 NPs (with 1 mg/kg of ICG) were injected intravenously into nude mice bearing unilateral HCC-LM3 tumors, and tumor biodistribution was recorded with an in vivo imaging system (IVIS Lumina, PerkinElmer, USA). Twenty-four hours after injection, the spleen, heart, lungs, kidney, liver and tumor were dissected, and the fluorescence signals emitted from each sample were recorded. To evaluate the enrichment of nanodrugs after IRFA, IRFA was performed on one side of each nude mouse bearing bilateral HCC-LM3 tumors, and sham RFA was performed on the other side. Seven days after IRFA, ICG@ZIF-8 NPs were injected, and the fluorescence signals of the bilateral tumors were recorded. The excitation wavelength was 740 nm.

### Immunohistochemistry staining

Tumor tissues were fixed in paraformaldehyde, embedded in paraffin, and cut into 3.5-mm-thick sections. After dewaxing, hydration and antigen retrieval, the tissue sections were incubated overnight with primary antibodies (anti-E-cadherin and anti-N-cadherin) at 4 °C and then incubated with secondary antibodies (MaxVision, Fujian, China) at room temperature for 1 h. After incubation with 3,3′-diaminobenzidine and hematoxylin, the tissues and nuclei were visualized under a microscope.

### Statistical analysis

GraphPad Prism 7.0 software (GraphPad Software, San Diego, CA) was used for data analysis. The quantitative data are expressed as the means ± standard deviation. In vitro experiments were repeated at least twice, and each in vivo experimental group included at least three mice. Statistical analysis of differences between two groups was performed with unpaired Student’s *t* test or a nonparametric test. Statistical analysis of differences among multiple groups was performed by ANOVA or rank sum test. A *P* value < 0.05 was recognized as significant.

## Supplementary Information

**Additional file 1: Figure S1.** Viability of Hep3B and SMMC7721 cells after treatment with a series of temperatures for 15 min. **, P < 0.01; ***, P < 0.001. **Figure S2.** Morphology of Hep3B and SMMC7721 cells 3 days after exposure to 37, 42, 44, 46, and 48 °C for 15 min. Black arrowhead, spindle shapes. Blue arrowhead, vacuolar changes. Scale bar, 1000 µm. **Figure S3.** The quantified number of tubule nodes and total branching length of HUVECs after coculture with or without the supernatant of sublethally heated Hep3B and SMMC7721 cells (n = 3). *, P < 0.05. **Figure S4.** (**A**) Establishment of IRFA subcutaneous tumor model under ultrasound guidance. Red arrow, RF electrode. (**B**) Bioluminescence imaging of subcutaneous tumors before or after IRFA. **Figure S5.** HE staining and immunohistochemical staining of CD34 in tumor tissues 21 days after IRFA or sham IRFA. Black arrow, blood vessel. Black dotted line, boundary between necrosis and residual cancer. Blue arrow, necrosis. Scale bar, 50 μm. **Figure S6.** Particle size distribution of ZIF-8 (A) and As@ZIF-8/PEG (B) as determined by TEM. **Figure S7.** Cellular uptake of FITC@ZIF-8 NPs by Hep3B cells at 40 min, 4 h and 8 h. Scale bar, 1000 µm. **Figure S8.** Colony formation of sublethally heated Hep3B and SMMC7721 cells after incubation with free ATO, As@ZIF-8 NPs or ZIF-8 NPs for 24 h. **Figure S9.** Quantitative analysis chart of living/dead cell double staining of sublethally heated Hep3B and SMMC7721 cells after the indicated treatment. **, P < 0.01. **Figure S10.** Apoptosis rates of sublethally heated Hep3B and SMMC7721 cells after incubation with free ATO, As@ZIF-8 NPs or ZIF-8 NPs. **Figure S11.** Healing curve of Hep3B (**A**) and SMMC7721 cells (**B**) after incubation with free ATO, As@ZIF-8 NPs and ZIF-8 NPs for 24 h. *, P < 0.05. **Figure S12.** Schematic diagram of ICG@ZIF-8/PEG preparation. **Figure S13.** Schematic diagram of the in vivo fluorescence imaging experiment. **Figure S14.** Antitumor rate obtained from the mice receiving different treatments (n = 5). ***, P < 0.001. **Figure S15.** Mouse weight curves during treatment with free ATO, As@ZIF-8 NPs or ZIF-8 NPs.

## Data Availability

All data generated or analyzed during this study are included in this published article and its additional files.

## Abbreviations

HCC: Hepatocellular carcinoma
EMT: Epithelial–mesenchymal transition
ZIF-8: Zeolitic imidazolate framework-8
ATO: Arsenic trioxide
IRFA: Insufficient radiofrequency ablation
RFA: Radiofrequency ablation
IC_50_: Half-maximal inhibitory concentration
EPR effect: Enhanced permeability and retention effect
MOFs: Metal–organic frameworks
NPs: Nanoparticles
PV: Percentage of blood vessels
HUVECs: Human umbilical vein endothelial cells
PEG: Polyethylene glycol
ICG: Indocyanine green
FITC: Fluorescein isothiocyanate
HE staining: Hematoxylin and eosin staining
EMT-TFs: EMT-inducing transcription factors
VEGF: Vascular endothelial growth factor
P/S: Penicillin/streptomycin
