# Correction to: Augmented EPR effect post IRFA to enhance the therapeutic efficacy of arsenic loaded ZIF-8 nanoparticles on residual HCC progression

**DOI:** 10.1186/s12951-022-01386-w

**Published:** 2022-04-02

**Authors:** Xuehua Chen, Yongquan Huang, Hui Chen, Ziman Chen, Jiaxin Chen, Hao Wang, Dan Li, Zhongzhen Su

**Affiliations:** 1 Department of Ultrasound, Fifth Affiliated Hospital of Sun Yat-sen University, Zhuhai, 519000 Guangdong China; 2 Guangdong Provincial Key Laboratory of Biomedical Imaging and Guangdong Provincial Engineering Research Center of Molecular Imaging, Fifth Affiliated Hospital of Sun Yat-sen University, Zhuhai, 519000 Guangdong China; 3 Fine Chemical Industry Research Institute, School of Chemistry, Sun Yat-sen University, Guangzhou, 510275 Guangdong China

## Correction to: J Nanobiotechnol 20:34 (2022) https://doi.org/10.1186/s12951-021–01161-3

Following publication of the original article [1], the authors reported that Fig. 4C was incorrect. The corrected Fig. 4 and the figure caption are given below. The corrections do not affect the results and conclusions. All authors agree to these corrections and apologize for these errors.

**Fig. 4:**
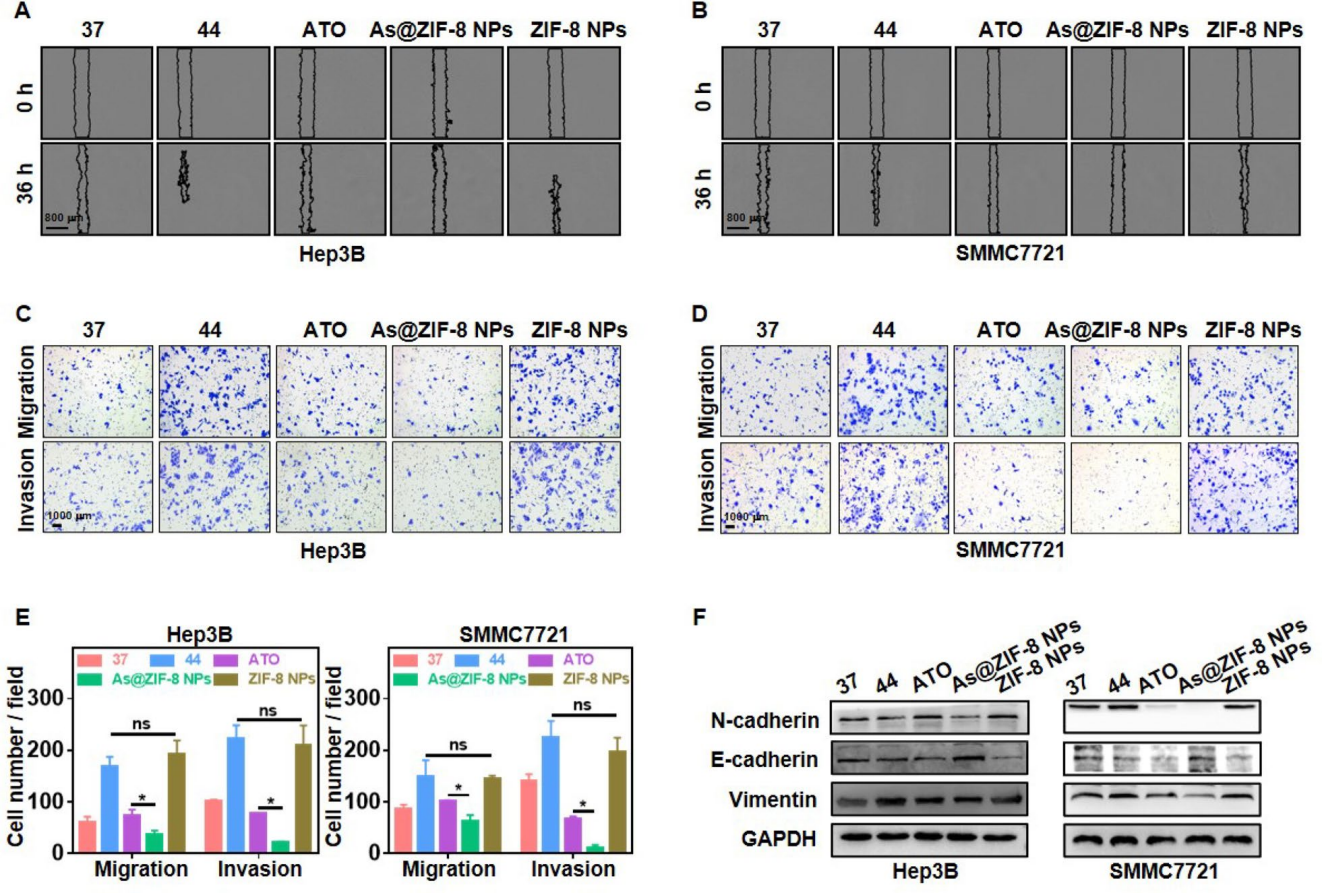
As@ZIF-8 NPs inhibited the invasion, migration and EMT of sublethally heated cells in vitro. Wound healing assay of sublethally heated Hep3B (**A**) and SMMC7721 cells (**B**) after incubation with free ATO, As@ZIF-8 NPs and ZIF-8 NPs for 24 h. Scale bar, 800 µm. Representative images of the migration and invasion of sublethally heated Hep3B (**C**) and SMMC7721 cells (**D**) after the indicated treatment and the related quantitative analysis chart (**E**). Scale bar, 1000 µm. (**F**) Protein expression of EMT markers N-cadherin, vimentin and E-cadherin in Hep3B and SMMC7721 cells after the indicated treatment. (ns, no statistical difference; *, P < 0.05)

## References

[1] Chen X, Huang Y, Chen H, Chen Z, Chen J, Wang H, Li D, Su Z (2022). Augmented EPR effect post IRFA to enhance the therapeutic efficacy of arsenic loaded ZIF-8 nanoparticles on residual HCC progression. J Nanobiotechnol.

