# Supplementary material for: Augmented EPR effect post IRFA to enhance the therapeutic efficacy of arsenic loaded ZIF-8 nanoparticles on residual HCC progression
Source: J Nanobiotechnology. 2022 Jan 15;20:34. doi: 10.1186/s12951-021-01161-3 (PMC8760822; doi:10.1186/s12951-021-01161-3)
Supplement: Supplementary file 1 — Additional file 1: Figure S1. Viability of Hep3B and SMMC7721 cells after treatment with a series of temperatures for 15 min. **, P < 0.01; ***, P < 0.001. Figure S2. Morphology of Hep3B and SMMC7721 cells 3 days after exposure to 37, 42, 44, 46, and 48 °C for 15 min. Black arrowhead, spindle shapes. Blue arrowhead, vacuolar changes. Scale bar, 1000 µm. Figure S3. The quantified number of tubule nodes and total branching length of HUVECs after coculture with or without the supernatant of sublethally heated Hep3B and SMMC7721 cells (n = 3). *, P < 0.05. Figure S4. (A) Establishment of IRFA subcutaneous tumor model under ultrasound guidance. Red arrow, RF electrode. (B) Bioluminescence imaging of subcutaneous tumors before or after IRFA. Figure S5. HE staining and immunohistochemical staining of CD34 in tumor tissues 21 days after IRFA or sham IRFA. Black arrow, blood vessel. Black dotted line, boundary between necrosis and residual cancer. Blue arrow, necrosis. Scale bar, 50 μm. Figure S6. Particle size distribution of ZIF-8 (A) and As@ZIF-8/PEG (B) as determined by TEM. Figure S7. Cellular uptake of FITC@ZIF-8 NPs by Hep3B cells at 40 min, 4 h and 8 h. Scale bar, 1000 µm. Figure S8. Colony formation of sublethally heated Hep3B and SMMC7721 cells after incubation with free ATO, As@ZIF-8 NPs or ZIF-8 NPs for 24 h. Figure S9. Quantitative analysis chart of living/dead cell double staining of sublethally heated Hep3B and SMMC7721 cells after the indicated treatment. **, P < 0.01. Figure S10. Apoptosis rates of sublethally heated Hep3B and SMMC7721 cells after incubation with free ATO, As@ZIF-8 NPs or ZIF-8 NPs. Figure S11. Healing curve of Hep3B (A) and SMMC7721 cells (B) after incubation with free ATO, As@ZIF-8 NPs and ZIF-8 NPs for 24 h. *, P < 0.05. Figure S12. Schematic diagram of ICG@ZIF-8/PEG preparation. Figure S13. Schematic diagram of the in vivo fluorescence imaging experiment. Figure S14. Antitumor rate obtained from the mice receiving different treatments (n [file 12951_2021_1161_MOESM1_ESM.doc]

Augmented EPR effect post IRFA to enhance the therapeutic efficacy of arsenic loaded ZIF-8 nanoparticles on residual HCC progression

*Xuehua Chen ^12‡^, Yongquan Huang ^12‡^, Hui Chen ^12^, Ziman Chen ^1^, Jiaxin Chen ^1^, Hao Wang ^3*^, Dan Li ^2*^, Zhongzhen Su ^12*^*

1. Department of Ultrasound, Fifth Affiliated Hospital of Sun Yat-sen University, Zhuhai, Guangdong Province, 519000, China.

2. Guangdong Provincial Key Laboratory of Biomedical Imaging and Guangdong Provincial Engineering Research Center of Molecular Imaging, Fifth Affiliated Hospital of Sun Yat-sen University, Zhuhai, Guangdong Province, 519000, China.

3. Fine Chemical Industry Research Institute, School of Chemistry, Sun Yat-sen University, Guangzhou, Guangdong Province, 510275,China.

‡These authors contributed equally

***Corresponding authors:

[suzhzh3@mail.sysu.edu.cn](mailto:suzhzh3@mail.sysu.edu.cn)

[lidan25@mail.sysu.edu.cn](mailto:lidan25@mail.sysu.edu.cn)

haowangfz@foxmail.com


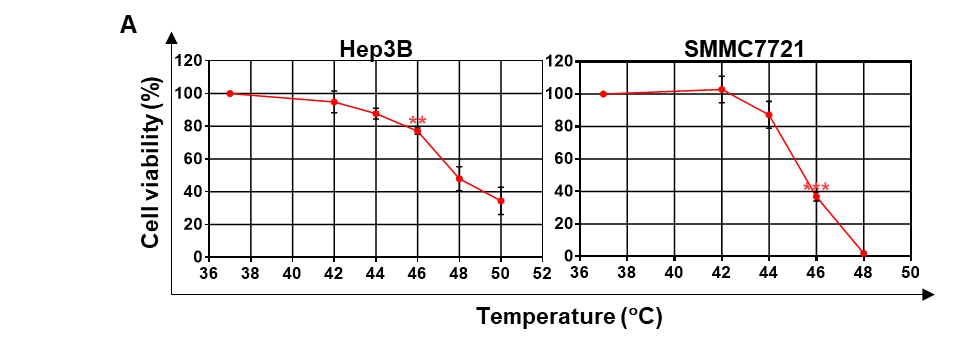


**Figure S1**. Viability of Hep3B and SMMC7721 cells after treatment with a series of temperatures for 15 min. **, P < 0.01; ***, P < 0.001.


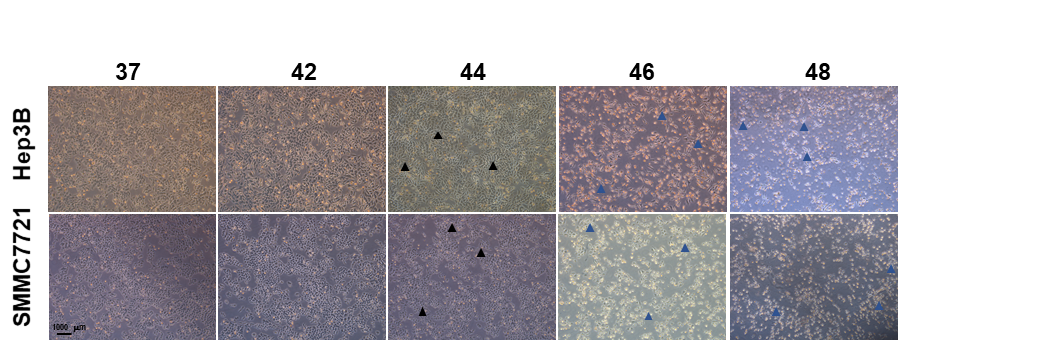


**Figure S2.** Morphology of Hep3B and SMMC7721 cells 3 days after exposure to 37, 42, 44, 46, and 48 ℃ for 15 min. Black arrowhead, spindle shapes. Blue arrowhead, vacuolar changes. Scale bar, 1000 µm.


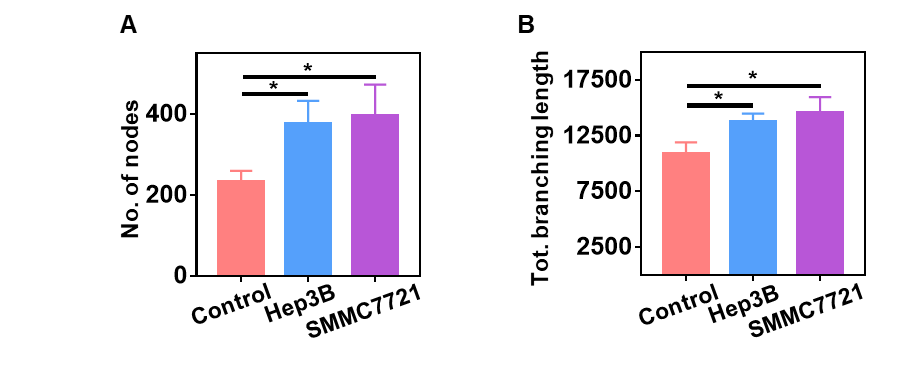


**Figure S3**. The quantified number of tubule nodes and total branching length of HUVECs after coculture with or without the supernatant of sublethally heated Hep3B and SMMC7721 cells (n = 3). *, P < 0.05.


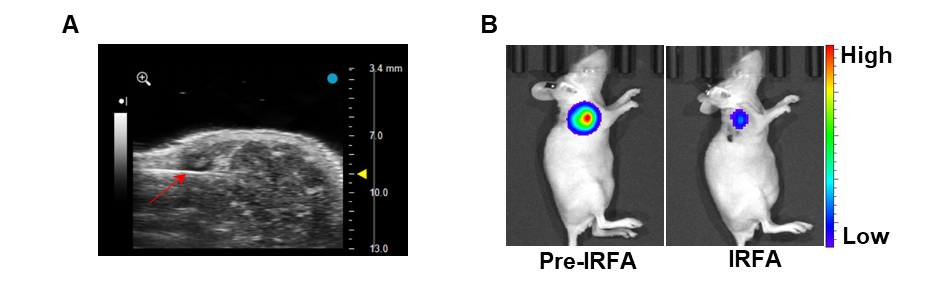


**Figure S4.** (**A**) Establishment of IRFA subcutaneous tumor model under ultrasound guidance. Red arrow, RF electrode. (**B**) Bioluminescence imaging of subcutaneous tumors before or after IRFA.


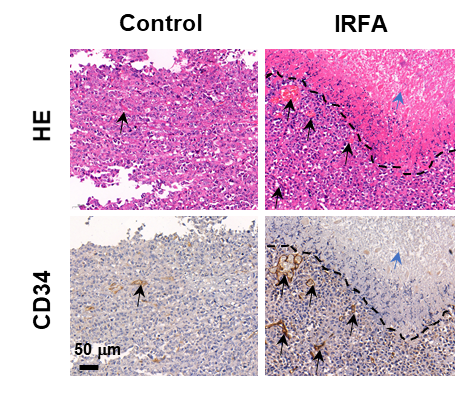


**Figure S5.** HE staining and immunohistochemical staining of CD34 in tumor tissues 21 days after IRFA or sham IRFA. Black arrow, blood vessel. Black dotted line, boundary between necrosis and residual cancer. Blue arrow, necrosis. Scale bar, 50 μm.


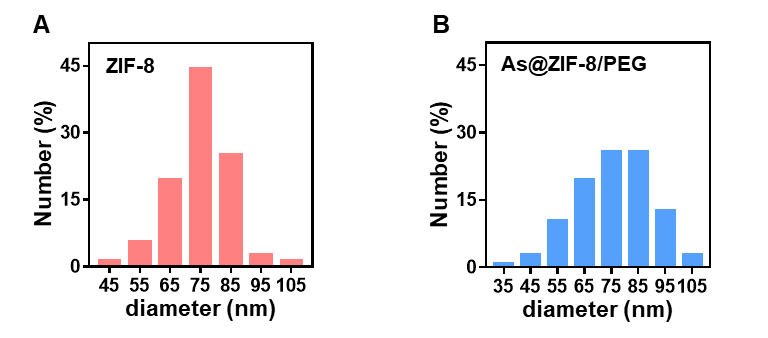


**Figure S6.** Particle size distribution of ZIF-8 (A) and As@ZIF-8/PEG (B) as determined by TEM.


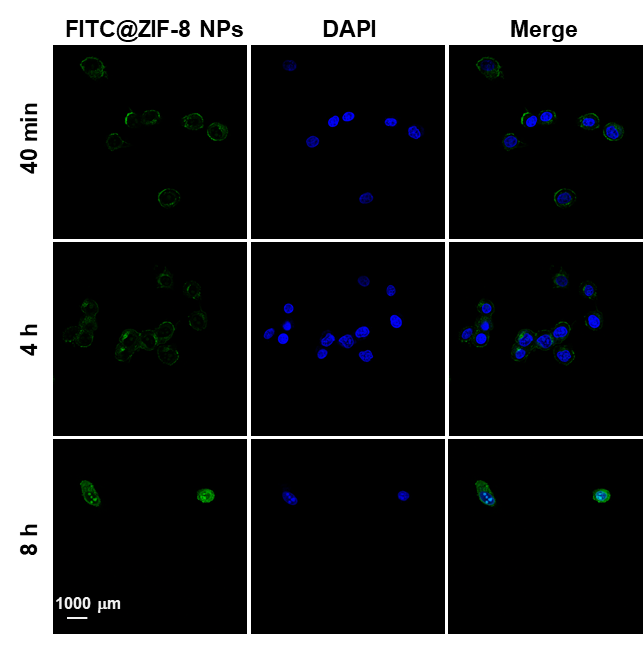


**Figure S7**. Cellular uptake of FITC@ZIF-8 NPs by Hep3B cells at 40 min, 4 h and 8 h. Scale bar, 1000 µm.

**
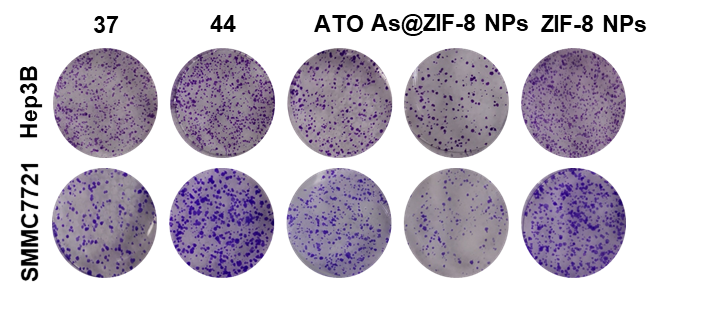
**

**Figure S8**. Colony formation of sublethally heated Hep3B and SMMC7721 cells after incubation with free ATO, As@ZIF-8 NPs or ZIF-8 NPs for 24 h.


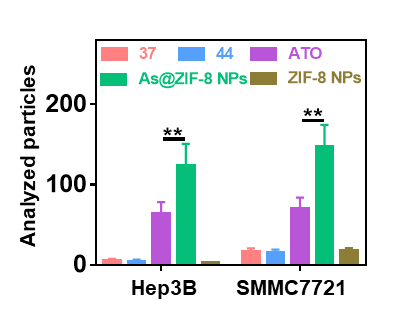


**Figure S9**. Quantitative analysis chart of living/dead cell double staining of sublethally heated Hep3B and SMMC7721 cells after the indicated treatment. **, P < 0.01.


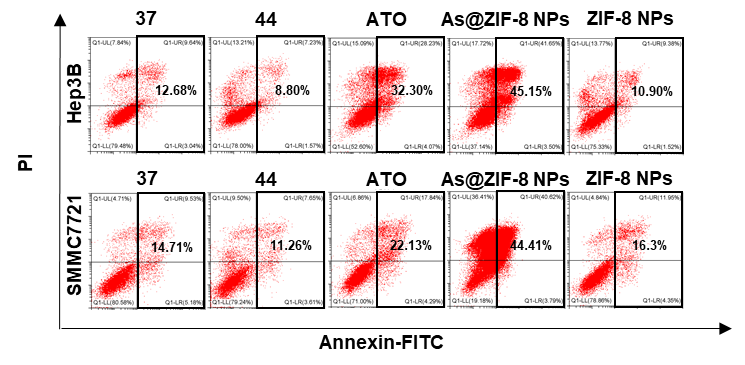


**Figure S10**. Apoptosis rates of sublethally heated Hep3B and SMMC7721 cells after incubation with free ATO, As@ZIF-8 NPs or ZIF-8 NPs.


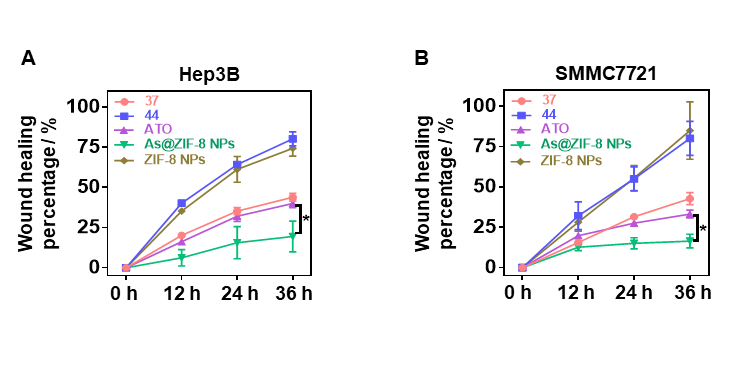


**Figure S11**. Healing curve of Hep3B (**A**) and SMMC7721 cells (**B**) after incubation with free ATO, As@ZIF-8 NPs and ZIF-8 NPs for 24 h. *, P < 0.05.


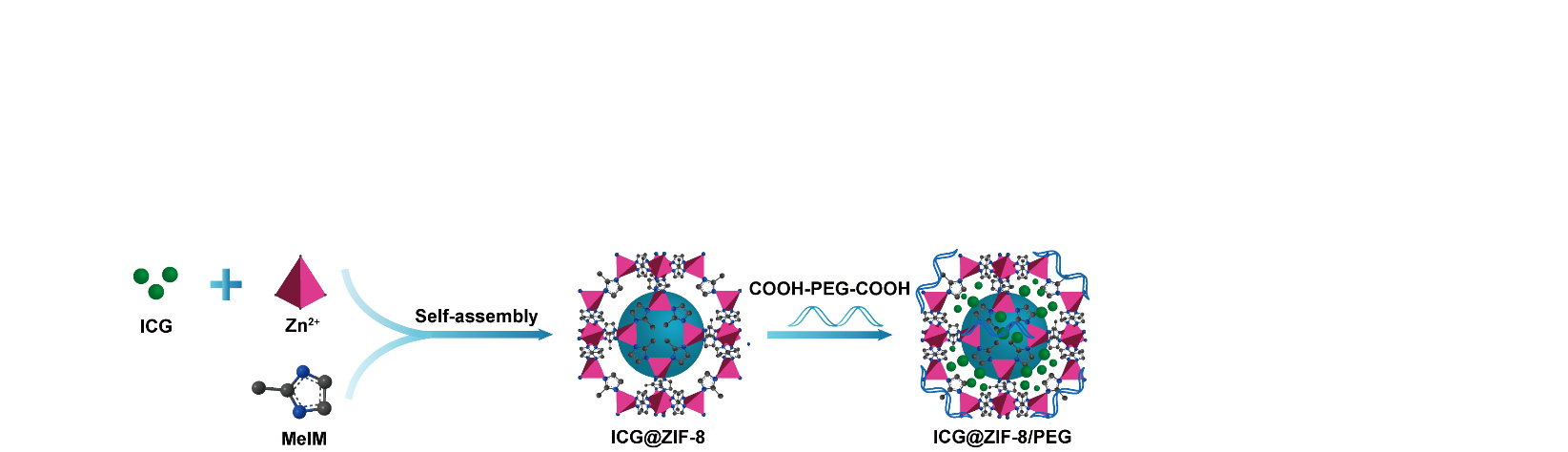


**Figure S12**. Schematic diagram of ICG@ZIF-8/PEG preparation.


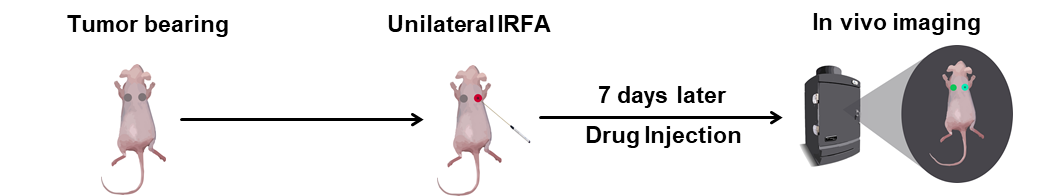


**Figure S13**. Schematic diagram of the in vivo fluorescence imaging experiment.

**Figure S14**. Antitumor rate obtained from the mice receiving different treatments (n = 5). ***, P < 0.001.


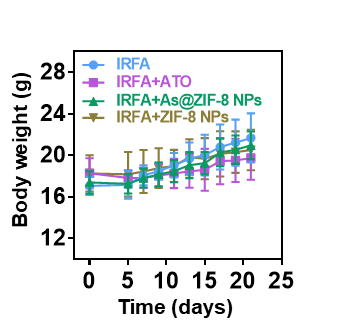


**Figure S15**. Mouse weight curves during treatment with free ATO, As@ZIF-8 NPs or ZIF-8 NPs.
